# Rigorous validation of graph-based network analysis reappraises biologically coherent ADHD-associated transcriptomic modules in peripheral blood

**DOI:** 10.1371/journal.pone.0359656

**Published:** 2026-10-05

**Authors:** Nehal M. Ali

**Affiliations:** School of Information Technology, Newgiza University, Cairo, Egypt; Tianjin Medical University General Hospital, CHINA

## Abstract

**Background:**

Graph attention networks (GATs) are increasingly applied to transcriptomic data because they integrate gene-network structure while producing attention weights that are often interpreted as indicators of biological importance. However, whether attention-derived explanations reliably reflect biologically meaningful signals has received little systematic evaluation, particularly in the small, heterogeneous cohorts common in psychiatric transcriptomics.

**Methods:**

We systematically re-evaluated a GAT using peripheral blood RNA-sequencing data from 76 individuals (39 ADHD, 37 controls), including 16 discordant monozygotic twin pairs. To maximize analytical rigor, we implemented a leakage-aware pipeline, incorporating twin-aware group-stratified cross-validation, fold-wise gene selection, corrected transcript-to-gene mapping, and validation through repeated cross-validation, permutation testing, three complementary feature-importance methods, orthogonal differential-expression and pathway analyses, and data-quality controls.

**Results:**

Across 20 repeated cross-validation splits, GAT achieved a slightly higher mean AUC than a graph convolutional network (mean AUC 0.624 vs. 0.599) and outperformed five classical machine-learning models on a prespecified split. However, GAT performance was not statistically distinguishable from a rigorously matched permutation-derived null distribution (p = 0.327), while an exploratory sample-size calculation indicated that roughly twice the current sample size would be needed to detect an effect this size at conventional power. Independent validation analyses converged on the same conclusion: attention-derived gene rankings were significantly negatively correlated with both SHAP and permutation importance, whereas differential-expression analyses---including discordant twin-pair comparisons---and pathway enrichment identified no reproducible biological signals after multiple-testing correction. Recovery of established co-expression modules and the absence of blood-cell marker differences supported pipeline validity.

**Conclusions:**

Graph-based models may show modest predictive gains, but predictive performance and biological interpretation are distinct scientific questions. Our findings demonstrate that attention-derived importance should be regarded as hypothesis-generating rather than mechanistic evidence unless independently validated, illustrating why rigorous, multi-level validation is essential before biological conclusions are drawn from graph neural networks in transcriptomic research.

## Introduction

Attention-deficit/hyperactivity disorder is common in childhood and persists into adulthood for many affected individuals [1,2]. Because its diagnosis remains purely behavioral, there is longstanding interest in whether accessible peripheral measures, including blood gene expression, could eventually inform diagnosis, subtyping, or mechanistic understanding [3]. Blood transcriptomic studies of ADHD have so far relied on modest sample sizes, a constraint common across psychiatric transcriptomics, which has spurred interest in computational methods that can extract more signal from limited data. The picture is further complicated by growing evidence that ADHD is not a single homogeneous condition but a label spanning phenotypes, such as childhood-onset, persistent, and late-diagnosed presentations, that may differ meaningfully in their underlying biology [4,5]; we return to the implications of this heterogeneity in the Discussion.

Graph-based models are one such computational approach. Rather than treating each gene as an independent feature, they incorporate a co-expression or protein-interaction network directly, allowing predictions to draw on relationships between genes rather than expression values alone. Graph attention networks (GATs) [6] have drawn particular interest in genomics because their attention mechanism appears to offer something beyond prediction: a built-in signal of which genes, or which gene-gene relationships, the model considered most important. In practice, this has led some studies to treat high-attention genes as candidate biological findings in their own right.

That assumption deserves closer scrutiny. Outside genomics, work in natural language processing has raised a specific concern: attention weights reflect how a model routes information internally, which is not necessarily the same as which inputs actually drove its output [7]. Whether this concern extends to graph attention networks trained on small transcriptomic cohorts, where the ratio of model parameters to samples is often unfavorable and subtle sources of statistical leakage or technical confounding are easy to overlook, has not, to our knowledge, been directly tested with the kind of multi-method validation that would settle the question.

We set out to do exactly that. Using a peripheral blood RNA-sequencing dataset of ADHD cases and controls that includes a subset of discordant monozygotic twin pairs [8], a design that allows genetically controlled comparisons unavailable in typical case-control cohorts, we asked four linked questions. First, how does graph-based model performance compare with conventional machine-learning approaches once major sources of cross-validation leakage are addressed? Second, does any observed performance difference remain distinguishable from a rigorously constructed permutation-based null? Third, when the model’s attention weights are compared against alternative, perturbation-based measures of feature importance, do they agree, and if not, what does that imply for treating attention-derived gene rankings as biological findings? Fourth, are any gene-level or pathway-level differences detectable through conventional means, including a design that controls directly for genetic background?

A single theme organizes all four questions: how does our confidence in a biological interpretation change as we apply increasingly rigorous validation? We report the results of that process here, and argue that the validation itself, independent of whether it confirms or overturns any specific biological claim, is the more durable and broadly useful contribution of this work.

## Methods

### Analysis pipeline overview

Fig 1 summarizes the full analysis pipeline and, in particular, the boundary between operations computed once on the full cohort and operations repeated independently within each cross-validation fold, a distinction directly relevant to how feature-selection and sample-relatedness leakage were avoided throughout this study. Preprocessing (transcript-to-gene aggregation, replicate averaging, low-expression filtering, log2 normalization) is unsupervised and participant-agnostic: it applies the same fixed rule to every participant regardless of diagnostic label or eventual fold assignment, and is therefore computed exactly once on the full 76-participant cohort before any cross-validation split exists. Every subsequent step that involves a choice, specifically which genes are selected, how expression values are scaled, and which graph edges are constructed, is instead repeated independently within each of the 5 cross-validation folds, using only that fold’s training participants; validation participants for a given fold are not seen by any of these choices, only by the final evaluation step. The 76 participants are first partitioned into 5 folds using group-stratified cross-validation, in which each of the 16 discordant monozygotic twin pairs is treated as a single, indivisible unit, guaranteeing both twins in a pair always fall on the same side of the training/validation boundary; this constraint is checked programmatically after every fold split generated in this study. Full step-by-step detail, including the specific answer to whether gene selection was performed before or within cross-validation, is provided in S1 Methods.

**Fig 1:**
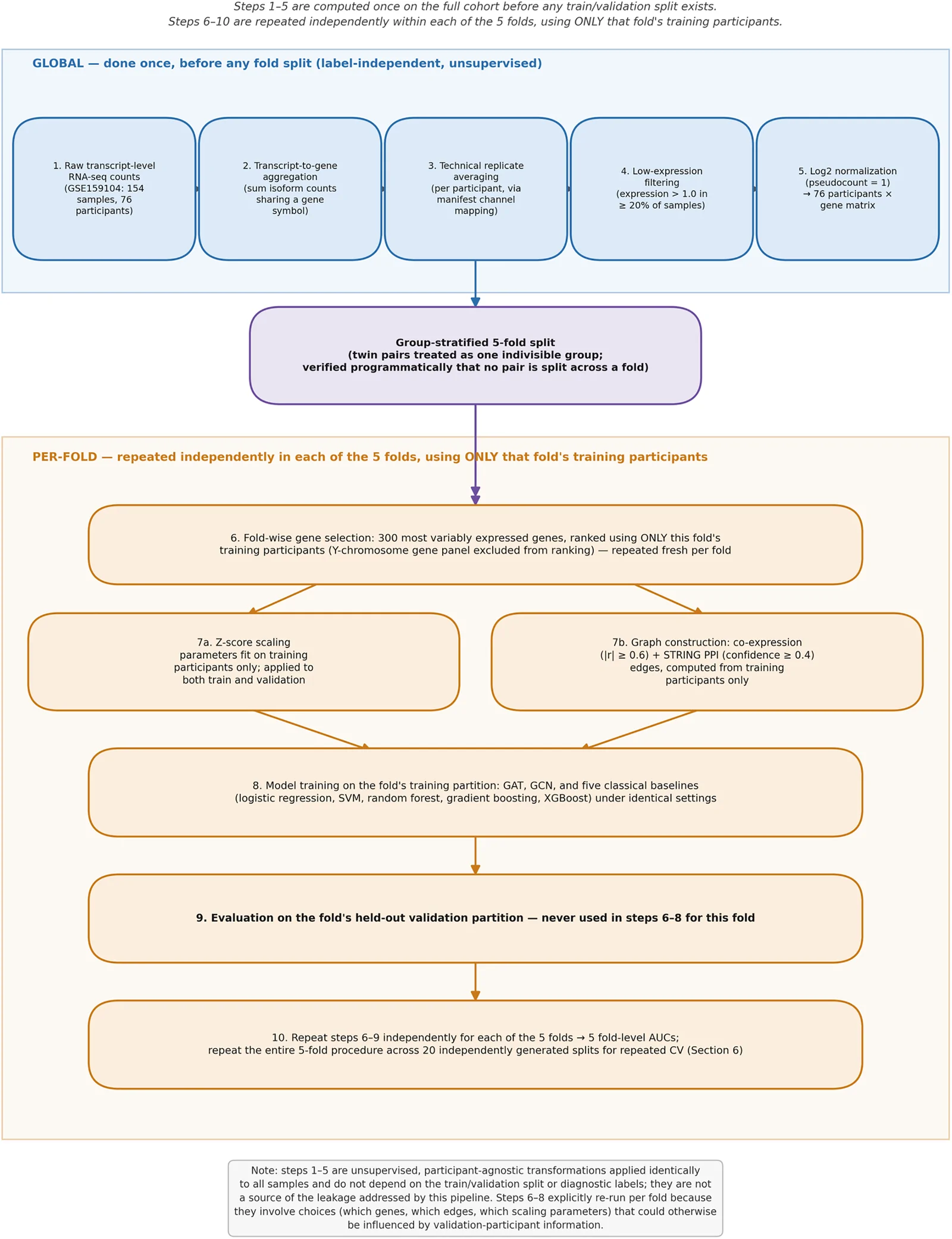
Analysis pipeline showing the boundary between global preprocessing and per-fold operations. Steps computed once on the full 76-participant cohort, before any train/validation split exists (global preprocessing: transcript-to-gene aggregation, replicate averaging, expression filtering, log2 normalization), versus steps repeated independently within each of the 5 cross-validation folds using only that fold’s training participants (fold-wise gene selection, feature scaling, and graph construction). Full step-by-step detail is provided in S1 Methods.

### Dataset and cohort characteristics

We analysed GSE159104 [8], deposited in the NCBI Gene Expression Omnibus, which was the primary publication reporting this dataset. Bruxel et al. [9] subsequently used these data in a multi-ancestry GWAS context; we cite both as relevant background. This dataset contains single-molecule whole-blood RNA-seq data from 154 samples representing 76 unique participants: 39 individuals with ADHD and 37 typically developing controls. The cohort embeds two complementary study designs: a discordant monozygotic twin sub-cohort (16 pairs, 32 participants) that controls simultaneously for genetic background, age, sex, and shared environment; and an independent case-control sub-cohort (44 unrelated participants). Twin pairing, diagnostic status, and sample-to-sequencing-channel mapping were taken directly from the study’s original sample manifest and verified programmatically to confirm complete and genuinely discordant pairing for all 16 pairs.

### Preprocessing and gene selection

The raw expression data are provided at the level of UCSC transcript identifiers rather than gene symbols; transcript-level counts were aggregated to the gene level by summing all transcript isoforms sharing the same gene symbol before any downstream analysis, since treating isoforms as independent features would artificially inflate variance-based gene ranking (Fig 1). Technical replicates were averaged per participant, low-expression genes were removed (expression > 1.0 in at least 20% of samples), and log2 normalization with a pseudocount of 1 was applied.

To reduce potential sex confounding in gene selection, given the well-documented male predominance of ADHD and the absence of individual-level sex metadata in the public GEO deposit of GSE159104, a fixed panel of Y-chromosome-encoded genes (including *UTY*, *KDM5D*, *EIF1AY*, *RPS4Y1*, and related Y-linked genes) was excluded prior to variance ranking; this step does not substitute for a fully sex-stratified analysis, which remains a limitation given the unavailability of sex annotations in the public dataset.

The 300 most variably expressed genes were retained as graph nodes, selected independently within each cross-validation fold’s training participants only (Fig 1; full step-by-step detail in S1 Methods). Variance-based selection was used rather than differential expression-based selection, given the limited per-fold sample size (approximately 60 training participants) for reliable false discovery rate control at the feature-selection stage.

### Cross-validation design addressing sample-relatedness and feature-selection Leakage

Because 32 of the 76 participants belong to genetically related twin pairs, standard cross-validation risks placing one twin in a fold’s training partition and their co-twin in the same fold’s validation partition, allowing a model to partially exploit genetic relatedness rather than learning generalizable signal. Group-stratified five-fold cross-validation addressed this by treating each twin pair as a single, indivisible group, with unrelated case-control participants forming their own singleton groups (Fig 1).

### Graph construction

For each cross-validation fold, co-expression edges were computed exclusively from that fold’s training partition: gene pairs with absolute Pearson correlation ≥ 0.6 received a co-expression edge weighted by absolute correlation, combined with knowledge-driven protein-protein interaction edges from STRING v11.5 (confidence ≥ 0.4) [5], with STRING confidence scores normalized to [0,1]. Resulting fold-specific graphs, built on the corrected gene-level expression data, contained approximately 2,900−5,500 unique edges across 300 gene nodes, a substantially sparser structure than graphs built on the pre-correction transcript-level data, consistent with many of the additional edges in the uncorrected version reflecting redundant isoform-to-isoform correlation rather than distinct gene-gene relationships. Participant-specific node features were z-score standardized expression values, with scaling parameters fit exclusively on each fold’s training participants and applied to validation participants (Fig 1).

### GAT and GCN architecture and training

A two-layer graph attention network was used for graph-level binary classification: the first layer applied two attention heads in concatenation mode (1 → 64 dimensions), and the second applied a single attention head in averaging mode (64 → 32 dimensions), both with ELU activation and dropout (p = 0.4). Global mean pooling aggregated node embeddings into a graph-level representation, followed by a two-layer classification head (32 → 16 → 2) with ReLU activation and dropout. A graph convolutional network (GCN) of matching hidden dimensionality (two convolutional layers) was evaluated identically, sharing the same graph structure as GAT but aggregating neighboring node information uniformly rather than through learned attention weights, a design intended to isolate the specific contribution of the attention mechanism from the more general contribution of incorporating graph structure at all.

Both edge-weighted architectures received the co-expression correlation strength and STRING confidence scores described above directly as edge attributes during training and inference, allowing both models to make use of the weighted graph structure rather than binary connectivity alone.

Both models were trained using the Adam optimizer (learning rate 0.001, L2 weight decay 1 × 10^-4^) for 150 epochs per fold, retaining the best-performing epoch by validation AUC on that fold’s own held-out validation partition; this same partition was used both for epoch selection and for the fold’s reported performance, without a separate inner validation split reserved exclusively for epoch selection, a point discussed further under Limitations. Five classical machine-learning baselines were evaluated under an identical cross-validation and fold-wise feature-selection protocol for direct comparison: logistic regression (L2-regularized), support vector machine (RBF kernel), random forest (200 estimators), gradient boosting (100 estimators), and XGBoost (100 estimators). Classical baselines received flat expression vectors without graph structure. Complete hyperparameter settings, random seeds, and software versions for all models are reported in S1 Table.

### Repeated cross-validation and paired GAT-GCN comparison

To assess whether the difference in performance between GAT and GCN reflected a stable property of the data rather than the particular fold assignment used, we generated 20 independent group-stratified five-fold splits and trained both models on each split, allowing a paired comparison of their mean cross-validated AUC across all 20 repeats (paired t-test).

### Permutation test

To test whether overall model performance exceeded chance expectation, we constructed a permutation-based null distribution. A fair comparison requires that the observed and null estimates be computed under identical conditions; the observed AUC was therefore computed as the mean of ten independently-trained models on the true diagnostic labels, using a fixed fold structure, fixed gene selection, and fixed graph topology, all under the same epoch budget subsequently used for the null distribution (150 permutations of shuffled diagnostic labels, trained under the identical fixed structure and epoch budget). The test was conducted at both a 50-epoch and a 100-epoch training budget to confirm that the result was not attributable to model under-convergence at the shorter budget. The empirical p-value was computed as the proportion of null permutations achieving AUC ≥ the matched observed value.

### Exploratory sample-size calculation

As an approximate, exploratory calculation rather than a formal power analysis, the observed cross-validated AUC (from the repeated cross-validation analysis above) was converted to an approximate Cohen’s *d* effect size using the relationship d = √2 × Φ^-1^(AUC), where Φ^-1^ is the inverse standard normal cumulative distribution function [10]. The sample size required for conventional 80% power at this effect size was estimated using a standard two-sample power formula. This calculation treats cross-validated AUC values as though independent across folds and repeats, which they are not, and is intended to give a rough, actionable sense of scale rather than a precise power estimate.

### Feature robustness: Attention weights, permutation importance, and SHAP

GAT attention weights were extracted from the saved best-epoch model for each fold in two complementary forms. First, per-edge attention differentials (mean value among ADHD participants minus mean value among controls) were computed, with genes appearing among the twenty highest-differential interactions in three or more of five folds classified as attention-robust (the prespecified robustness threshold; a lower threshold of two or more folds was used only for network visualization purposes, described below, to display broader network structure). Second, to enable a more granular, continuous comparison against the perturbation-based importance measures described below, a mean attention score was computed for each gene across all incident graph edges and all participants within each fold, without restriction to a fixed top-N list, and averaged across folds in which the gene was present.

Permutation importance was computed by shuffling each gene’s expression values among validation participants in turn, using the corresponding fold’s already-trained model, and recording the resulting reduction in validation AUC relative to the unshuffled baseline, averaged over 20 independent shuffles per gene per fold. SHAP values [11] were computed via a model-agnostic kernel explainer, with each fold’s trained model and fixed graph topology wrapped as a black-box function of per-participant gene expression; to remain within GPU memory constraints, synthetic perturbation batches generated by the explainer were processed in fixed-size chunks rather than as a single batch.

Agreement between the three methods was assessed by comparing each method’s twenty highest-ranked genes directly, and --- since top-list overlap is a coarse measure that can obscure genuine partial agreement or disagreement in rank ordering, by computing Spearman rank correlation between each pair of methods across their full common set of scored genes and at fixed top-N thresholds (100, 200, and 500 genes).

### Reference-module co-expression coherence and node ablation

Co-expression coherence of two predefined reference gene sets was assessed by computing mean within-set Pearson correlation, in each fold’s training partition, for two literature-derived reference gene sets: an immune/interferon-response module (*IFITM3*, *IFI44L*, *RSAD2*, *IFIT1*) and a platelet/cell-adhesion module (*ITGA2B*, *TNS1*, *TRIM58*), with r > 0.3 used as an illustrative coherence threshold drawn from weighted gene co-expression network analysis conventions [7]; this analysis evaluates coherence of two small, literature-derived gene sets (four and three genes, respectively) and does not constitute formal WGCNA-style module preservation testing. Node ablation was performed for each of these seven genes, where present in a given fold’s selected gene set, by zeroing the gene’s value in the input feature matrix, retraining the model from scratch on that fold, and recording the resulting change in validation AUC relative to the intact model.

### Orthogonal differential expression analyses

Independent of the graph-based model, conventional differential expression analysis (Welch’s t-test per gene, Benjamini-Hochberg false discovery rate correction) was conducted comparing ADHD and control participants, performed separately on the full 76-participant cohort and, given that 32 of these participants are members of related twin pairs and therefore not statistically independent observations, on the 44 unrelated case-control participants alone.

Within the 16 discordant twin pairs, a paired differential expression analysis was additionally conducted (paired t-test per gene, with the same false discovery rate correction, and Wilcoxon signed-rank testing of the top fifty candidate genes as a non-parametric sensitivity check given the modest number of pairs). Because monozygotic co-twins share essentially all germline genetic variation, this design controls for genetic background directly, in a manner not available to the case-control comparisons above.

### Pathway-level enrichment analysis

To test whether genes failing to converge across the three feature-importance methods might nonetheless implicate shared biological pathways, gene set enrichment analysis (pre-ranked, MSigDB Hallmark gene set collection [12,13]) was conducted separately on the full ranked gene list produced by each of the three methods (attention, permutation importance, SHAP), and the resulting significantly enriched pathways (false discovery rate < 0.25, a conventional threshold for exploratory pathway-level analysis) were compared across methods for overlap.

### Blood cell-type marker-expression analysis

Because whole-blood transcriptomic differences can, in principle, reflect shifts in relative blood cell-type proportions rather than within-cell-type regulatory differences, proxy marker-expression scores were calculated for each participant across six major blood cell types (neutrophils, T cells, B cells, natural killer cells, monocytes, and platelets; three literature-established marker genes per panel, averaged), rather than through a validated reference-based deconvolution algorithm. Mean marker-expression score per panel was compared between ADHD and control groups (Welch’s t-test), and correlation between each attention-robust gene’s expression and each cell-type marker score was examined as an indicator of potential non-specific technical covariation.

### Software

Analyses were conducted in Python using PyTorch and PyTorch Geometric (graph-based models), scikit-learn (classical baselines and cross-validation utilities), SHAP, gseapy, statsmodels, and scipy.

## Results

### Dataset quality

Principal component analysis of the log2-normalized, gene-aggregated expression data found no evidence of gross batch structure or outlier clustering: ADHD and control participants, and members of the twin and case-control sub-cohorts, were distributed without visible separation along the first two principal components (52.5% and 5.5% of total variance, respectively) (Fig 2).

**Fig 2:**
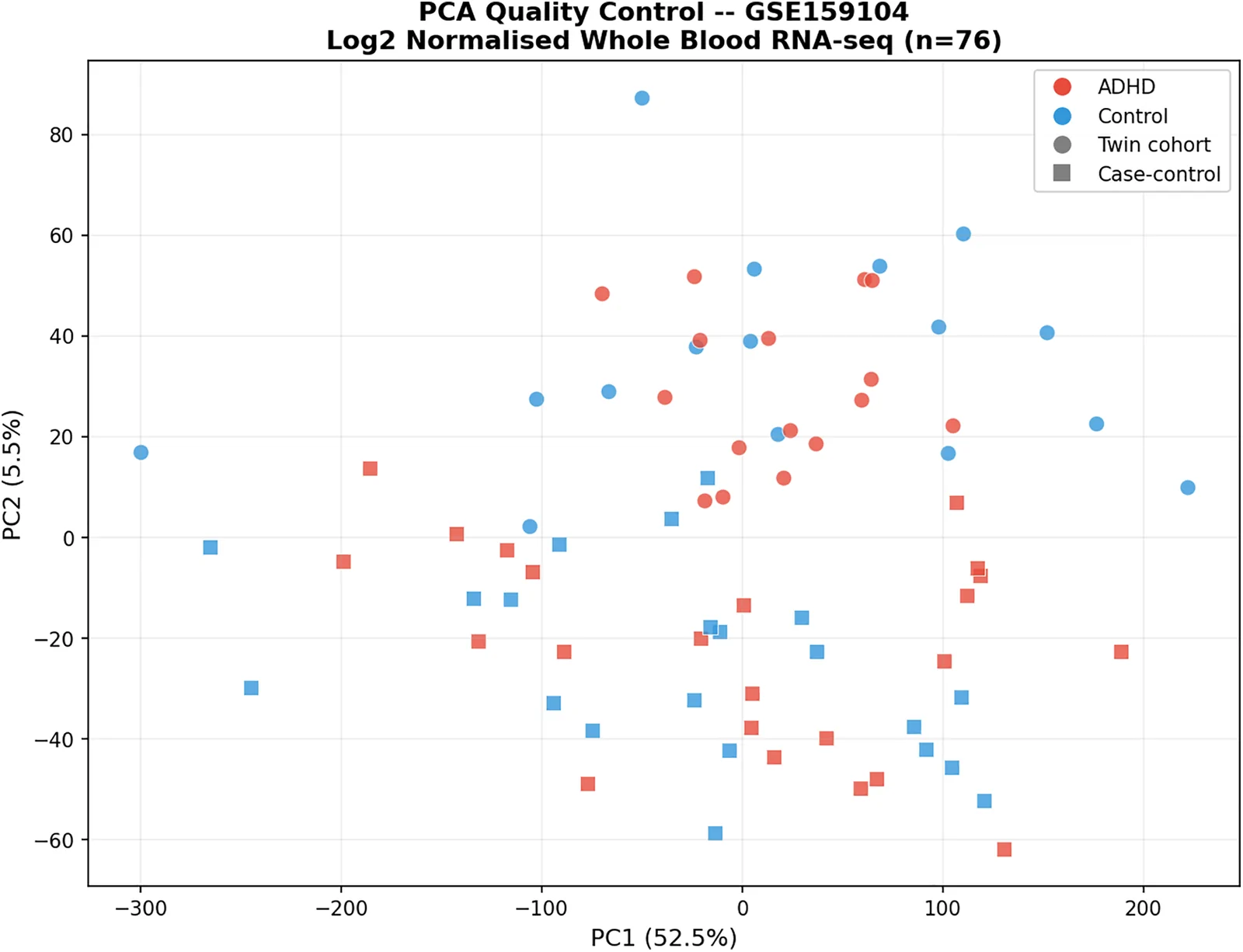
Principal component analysis of whole-blood transcriptomic data. PCA of log2-normalized expression values (n = 76 participants) shows no evidence of gross batch effects or outlier clustering by diagnostic group (ADHD, red; control, blue) or cohort design (twin sub-cohort, circles; case-control sub-cohort, squares). PC1 and PC2 explain 52.5% and 5.5% of total variance, respectively.

### Graph-based models showed modest predictive gains over classical machine-learning baselines

Under the revised cross-validation protocol addressing sample-relatedness and feature-selection leakage, both graph-based models achieved higher AUCs than the classical baselines evaluated on this prespecified split. The GAT achieved a mean AUC of 0.67 (SD 0.15) across five folds, compared with 0.42--0.53 for the five classical methods (logistic regression 0.42, SVM 0.53, random forest 0.52, gradient boosting 0.37, XGBoost 0.44); the GCN achieved a mean AUC of 0.57 (SD 0.14) (Fig 3). To assess whether this difference was stable rather than an artifact of a single data split, we repeated the GAT-versus-GCN comparison across 20 independently generated cross-validation splits. Averaged across these repeats, GAT achieved a mean AUC of 0.624 (SD 0.042, 95% CI 0.605--0.643) and GCN a mean AUC of 0.599 (SD 0.034, 95% CI 0.585--0.613); the paired difference (+0.025, 95% CI 0.001--0.047) reached nominal statistical significance (paired t-test, p = 0.049), with GAT outperforming GCN in 13 of 20 repeats. Given how close this result sits to the conventional significance threshold, and given the considerably larger apparent gap observed within any single data split (up to 0.15 AUC points in some individual folds), we treat this as a small and statistically fragile difference favoring attention-based aggregation, and rely on the repeated cross-validation estimate, not any single split, as the more trustworthy summary of model performance throughout the remainder of this paper.

**Fig 3:**
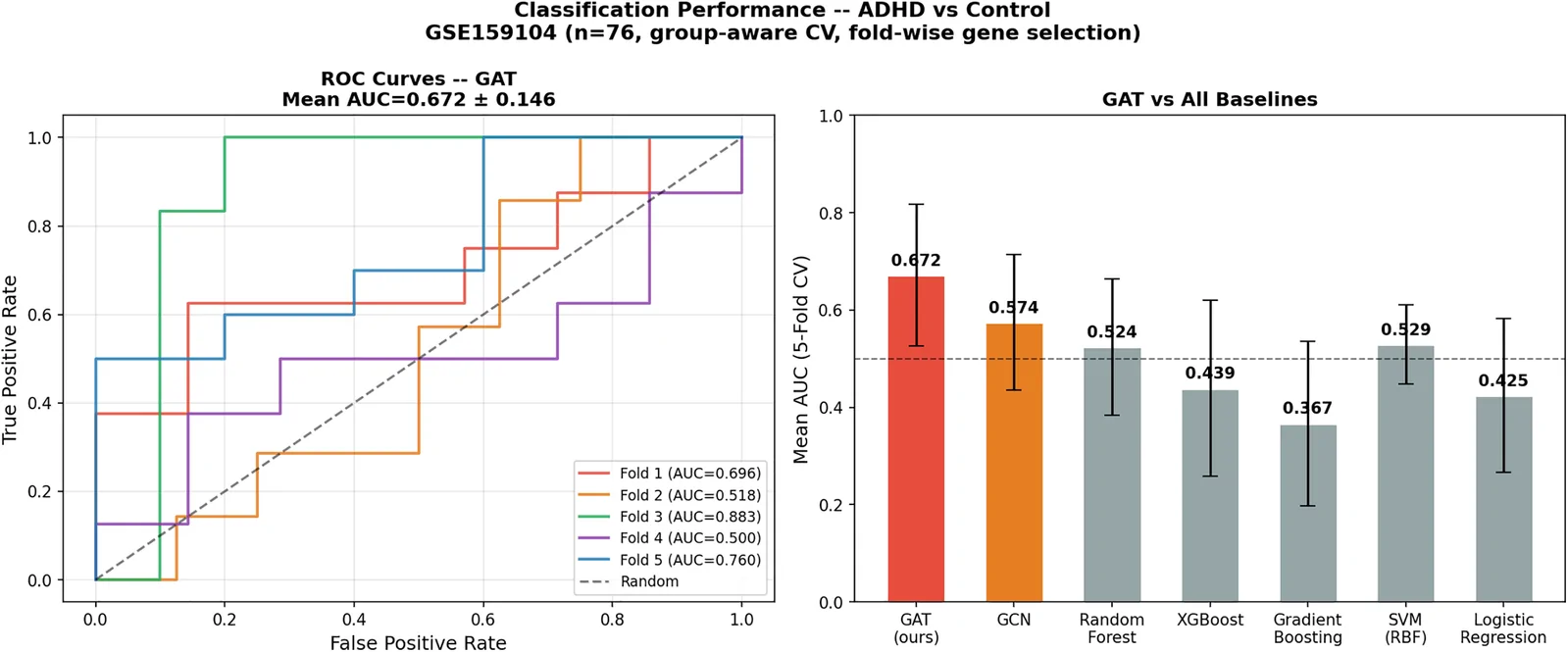
Classification performance of GAT compared with baseline models. Left: receiver operating characteristic (ROC) curves for the graph attention network (GAT) across five cross-validation folds (individual fold AUC values shown in the legend; mean AUC = 0.672 ± 0.146). Right: mean five-fold cross-validated AUC for GAT, the graph convolutional network (GCN) comparison model, and five classical machine-learning baselines, with error bars showing standard deviation across folds. The dashed line indicates chance-level performance (AUC = 0.5).

### Performance did not exceed a permutation-based null, and the study was underpowered to detect the observed effect

Comparing the observed AUC (mean of ten independently-trained models under a fixed fold structure, 0.644 ± 0.029) against a null distribution built from 150 label permutations trained under matched conditions (null mean 0.613 ± 0.072) found no significant difference (empirical p = 0.327). This result was consistent whether the underlying models were trained for 50 or 100 epochs, indicating that model under-convergence at the shorter budget was not the explanation.

An approximate, exploratory sample-size calculation offers relevant context. Treating the observed effect size (Cohen’s *d* ≈ 0.45, derived from the repeated cross-validation AUC of 0.624) as the population effect, a standard two-sample power formula indicated that approximately 159 participants (79 per group), roughly double the available cohort, would be required to detect an effect of this magnitude with conventional 80% power at this sample size; the estimated achieved power at n = 76 was approximately 49%. We report this as an approximation intended to contextualize the non-significant permutation result, not as a precise or independent confirmation of it.

### Attention-derived gene rankings were negatively correlated with perturbation-based importance measures

Comparing the twenty highest-ranked genes from attention weights, permutation importance, and SHAP found no genes shared across all three methods, and no genes shared between attention and either of the other two methods individually (permutation importance and SHAP shared two genes: *SMARCA4* and *SNORD12*) (Fig 4).

**Fig 4:**
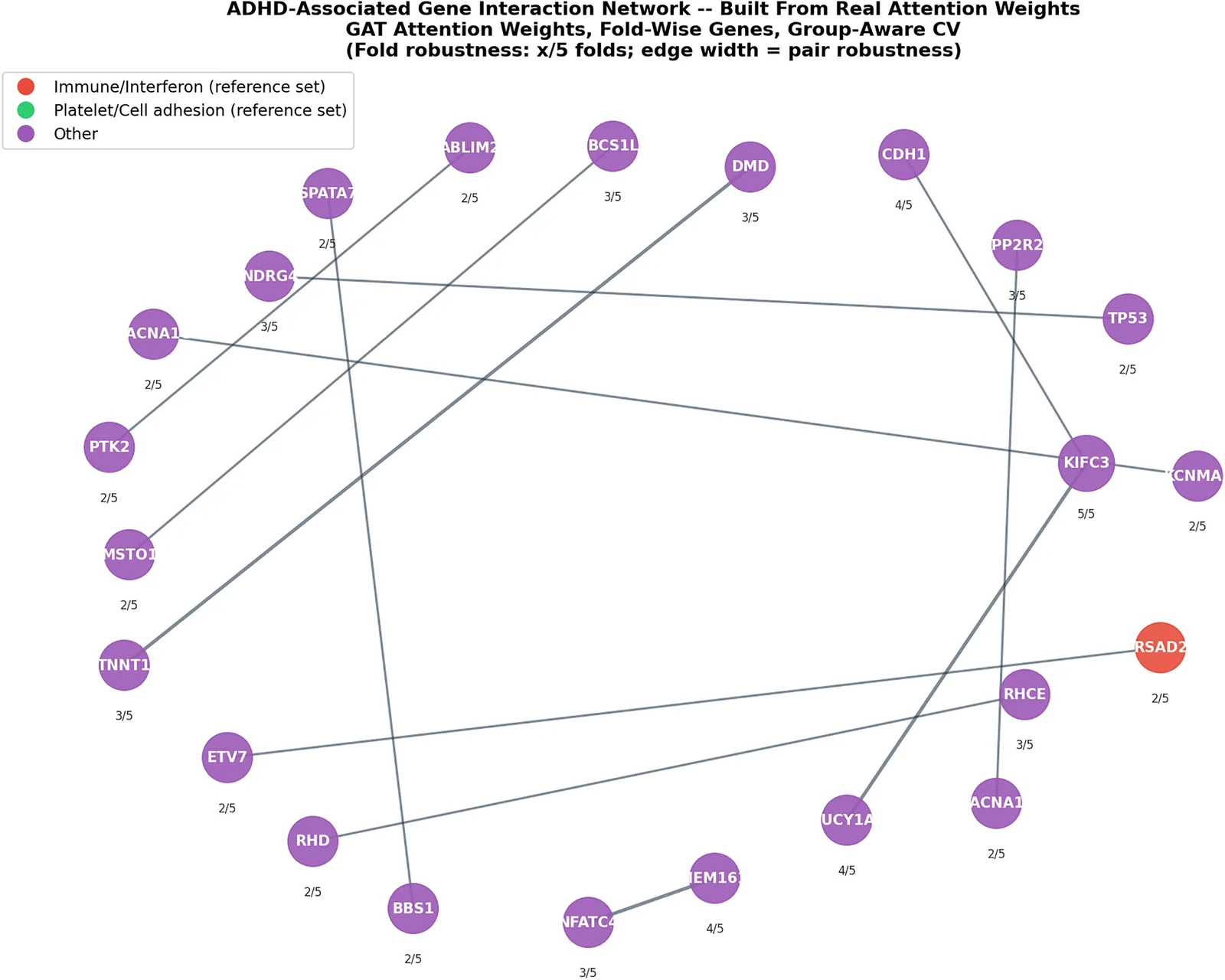
Gene interaction network derived from GAT attention weights. Network nodes represent genes appearing among the twenty highest attention-weighted interactions in at least two of five cross-validation folds (a relaxed threshold used here only to display broader network structure; the prespecified robustness threshold used elsewhere in this manuscript was three or more of five folds); edge width reflects the number of folds in which a given gene pair was jointly identified (labeled x/5). Node color indicates membership in the literature-derived immune/interferon or platelet/adhesion reference modules (red, green) versus genes outside these reference sets (purple). Only one literature-derived candidate gene (RSAD2) appears in the network, with low fold robustness (2/5) and a single connection.

Node ablation of the seven literature-derived candidate genes (*ITGA2B*, *IFITM3*, *IFI44L*, *RSAD2*, *IFIT1*, *TNS1*, *TRIM58*) found no gene producing a robust, positive AUC reduction consistent with meaningful predictive importance; two genes (*IFI44L*, *RSAD2*) produced a net AUC *increase* upon removal, and one gene (*IFITM3*) was present in only a single fold, precluding a reliable importance estimate for it specifically (Fig 5).

**Fig 5:**
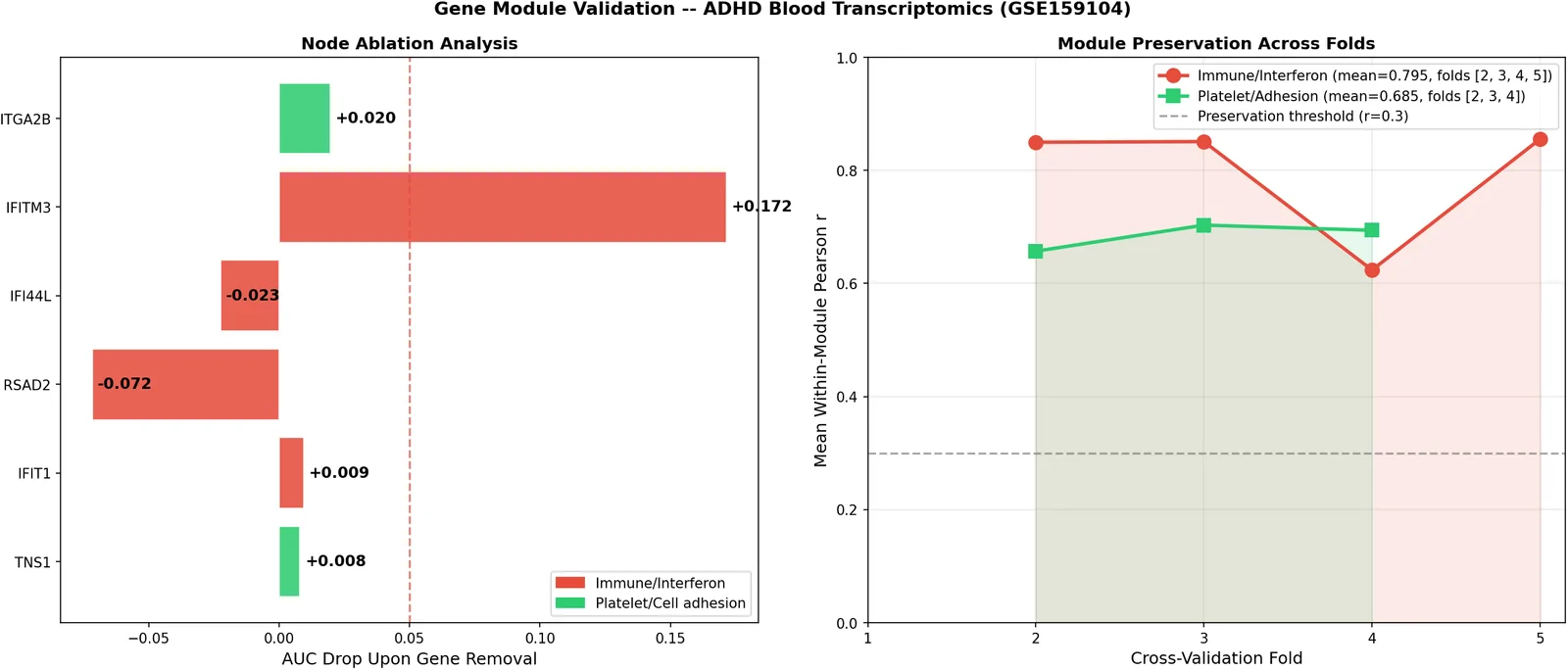
Node ablation analysis and cross-fold reference-module co-expression coherence. Left: change in validation AUC upon individually zeroing each of seven literature-derived candidate genes and retraining from scratch, colored by reference module membership (immune/interferon, red; platelet/adhesion, green). Right: mean within-set Pearson correlation for the immune/interferon and platelet/adhesion reference gene sets across cross-validation folds in which sufficient genes were available for testing; the dashed line indicates the illustrative coherence threshold (r = 0.3).

Even when restricted to an identical set of genes, SHAP and permutation importance rankings diverged substantially (Fig 6); for example, *TMEM68* showed high SHAP importance but a permutation-importance value indicating no positive contribution to model performance. Spearman rank correlation across the full set of 475 genes scored by all three methods (full correlation results in S2 Table) found attention-derived importance significantly negatively correlated with both permutation importance (ρ = −0.35, p < 0.0001; ρ = −0.57 restricted to each method’s top 100 genes) and SHAP (ρ = −0.22, p < 0.0001; ρ = −0.41 at top 100); permutation importance and SHAP showed a weak positive correlation with each other (ρ = 0.27, p < 0.0001 at the full-set level).

**Fig 6:**
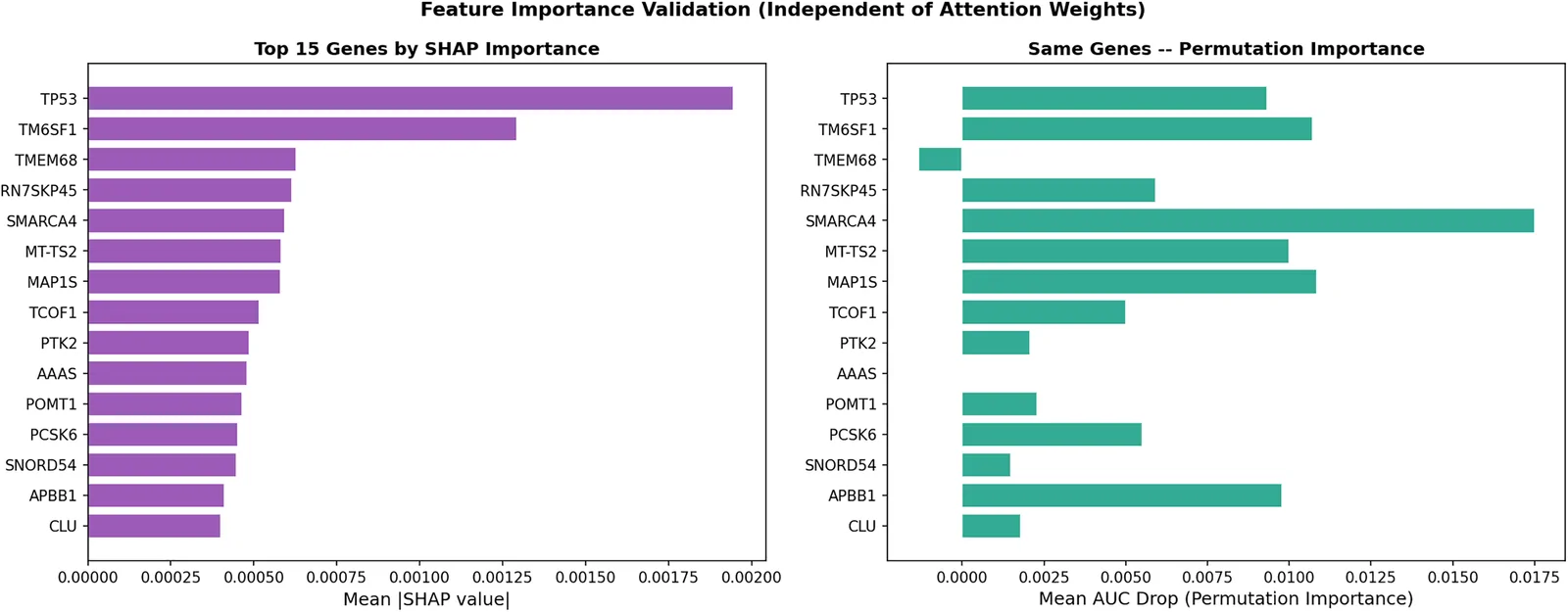
Independent validation of feature importance: SHAP versus permutation importance. Left: the fifteen genes with the highest mean absolute SHAP value, aggregated across cross-validation folds. Right: permutation importance (mean validation AUC drop upon shuffling) for the same fifteen genes, illustrating divergence between the two methods even when restricted to an identical gene set (for example, TMEM68 shows high SHAP importance but a permutation-importance value indicating no positive contribution to model performance).

Pathway-level enrichment analysis did not resolve this discordance: attention-ranked and permutation-importance-ranked genes yielded no pathways reaching FDR < 0.25, while SHAP-ranked genes implicated three pathways (DNA repair, E2F targets, p53 pathway) at this threshold, with no overlap with the other two methods even at the pathway level.

### No gene reached convergent significance across five complementary analytical approaches

Neither conventional differential expression run on the full 76-participant cohort, nor the same analysis restricted to the 44 statistically independent case-control participants, identified any gene reaching FDR < 0.05 or the more lenient FDR < 0.10 (0 genes in both analyses). The two versions showed limited agreement even with each other (2 of their respective twenty highest-ranked genes overlapping), illustrating the sensitivity of top-ranked gene lists to even modest changes in exactly which participants are included at this sample size. The paired analysis within the 16 discordant twin pairs, a design offering stronger control of genetic background but a smaller effective sample size than the case-control comparisons above, likewise identified no gene reaching FDR < 0.05 or FDR < 0.10 (Fig 7).

**Fig 7:**
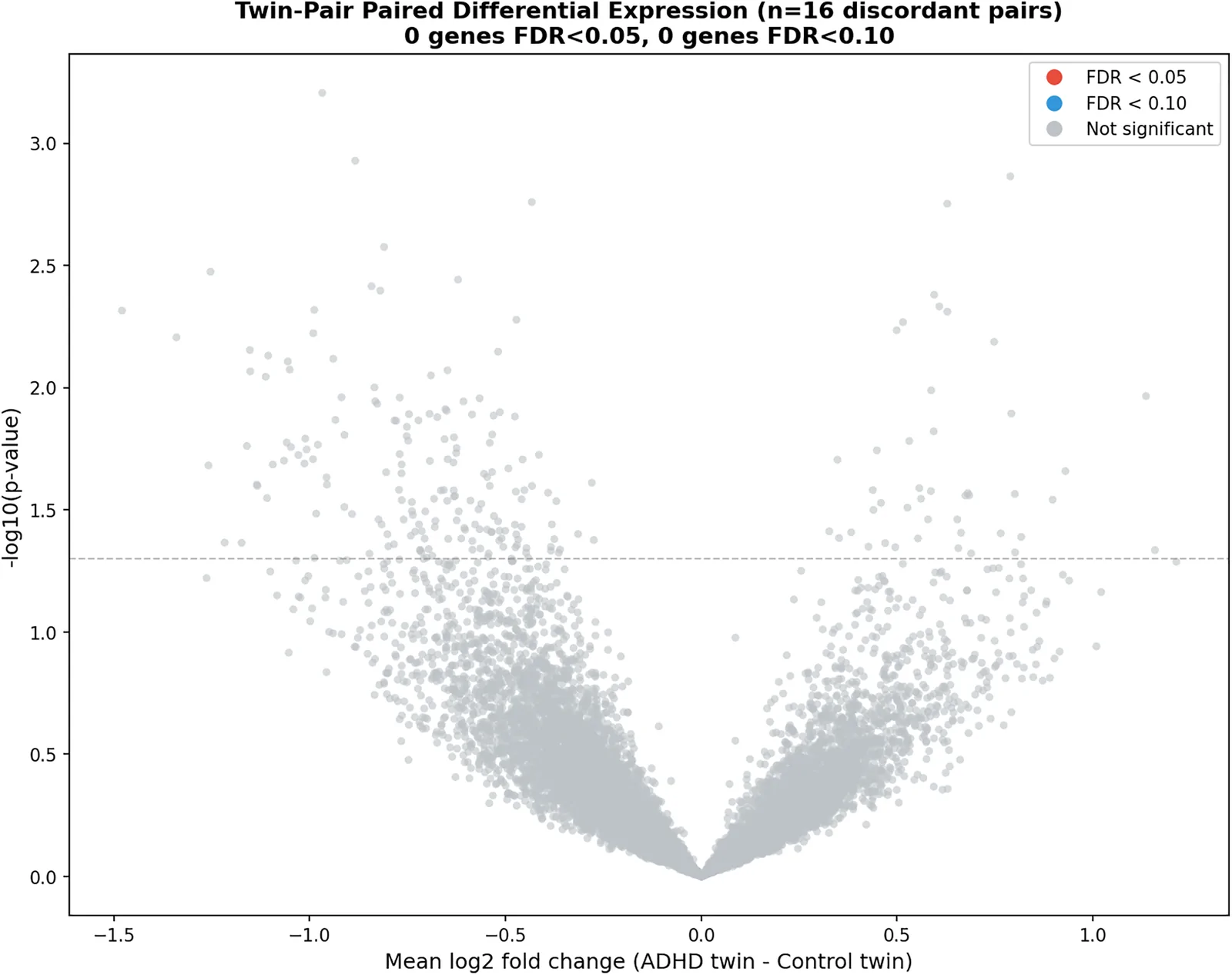
Paired differential expression within discordant monozygotic twin pairs. Volcano plot of paired differential expression testing (n = 16 discordant twin pairs; paired t-test per gene). No gene reached statistical significance after false discovery rate correction at either FDR < 0.05 or FDR < 0.10.

### Reference-module co-expression coherence and marker-based cell-type scores provided a limited positive control

Two small, predefined reference gene sets (an immune/interferon set and a platelet/adhesion set) showed strong within-set co-expression coherence in the folds where sufficient module genes were available (immune/interferon: mean r = 0.795, present in four of five folds; platelet/adhesion: mean r = 0.685, present in three of five folds), both exceeding the illustrative coherence threshold (r > 0.3).

Proxy marker-expression scores for six major blood cell types did not differ significantly between ADHD and control groups at the group level for any cell type examined (p = 0.38–0.84) (Fig 8). However, several of the genes most consistently highlighted by attention weights showed broad, non-specific positive correlation with multiple, biologically unrelated cell-type marker panels simultaneously, a pattern more consistent with a shared technical covariate, such as total RNA yield, than with genuine cell-type-specific biology, and one offering a plausible partial explanation for the discordance between attention-derived and perturbation-based importance measures reported above.

**Fig 8:**
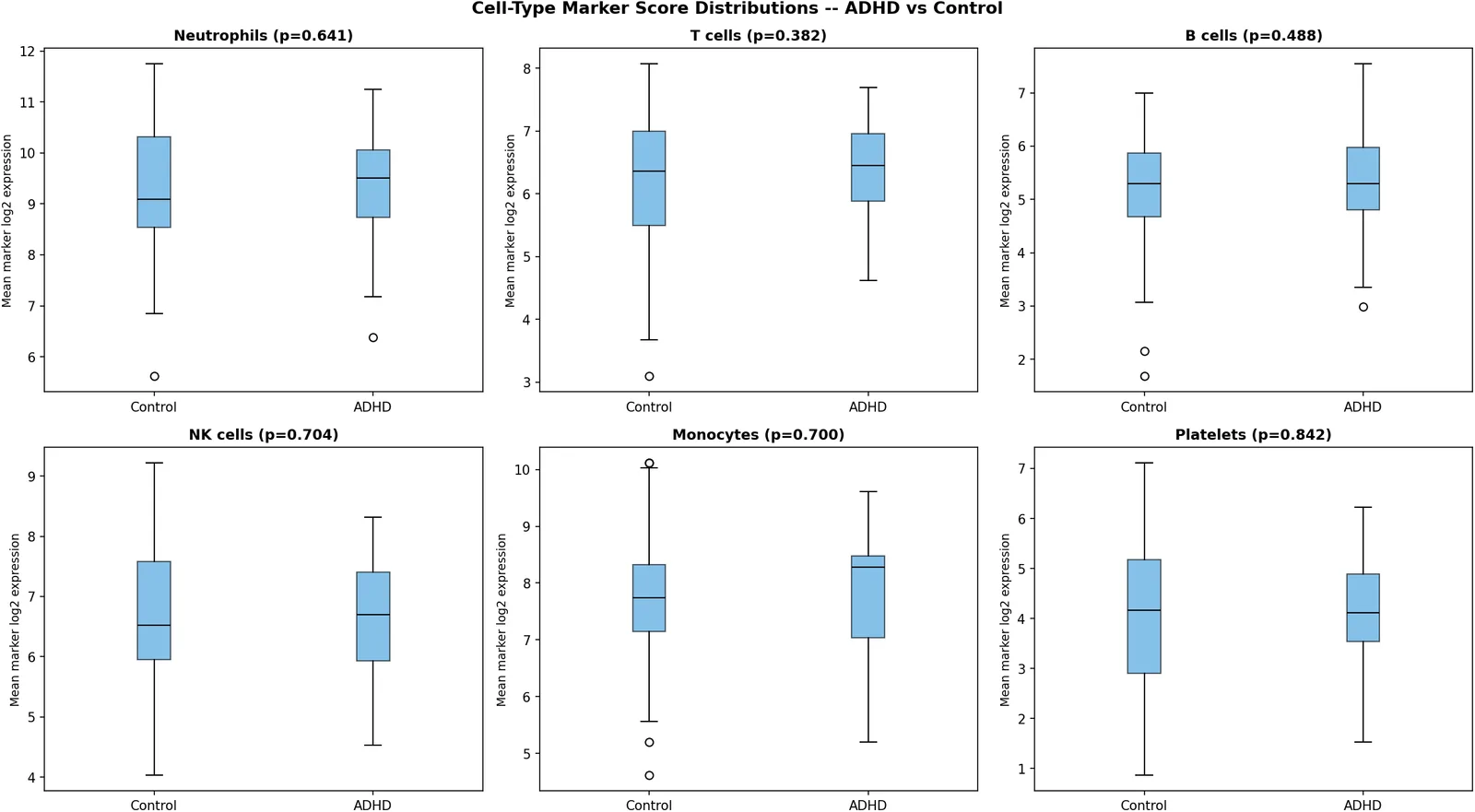
Cell-type marker score distributions by diagnostic group. Distribution of mean marker-gene expression (log2 scale) for six canonical blood cell-type panels (neutrophils, T cells, B cells, natural killer cells, monocytes, platelets), compared between ADHD and control participants (Welch’s t-test p-value shown per panel). No panel showed a statistically significant group difference.

## Discussion

This study did not set out simply to ask whether a graph-based model could classify ADHD from control participants using blood transcriptomic data. Rather, we asked whether the modest predictive performance observed under a cross-validation framework designed to address major sources of sample-relatedness and feature-selection leakage would withstand additional validation. The more consequential question was whether that performance, and the gene-level explanations the model appeared to offer alongside it, would hold up against a comprehensive set of independent validation checks [14]. Largely, they did not, and the pattern of what did and did not survive scrutiny is itself informative.

### Comparison of graph-based and conventional machine-learning performance

Network-based models achieved higher AUCs than the classical feature-based approaches evaluated on the prespecified split, while repeated cross-validation showed only a small difference between GAT and GCN [15]. That said, the advantage is neither large nor definitive. The GAT-versus-GCN comparison, which isolates whether the attention mechanism itself adds anything beyond graph structure generally, produced a paired difference whose confidence interval sat just above zero (p = 0.049). A difference this modest and this fragile statistically is better treated as an open question for future, adequately powered work than as a settled finding.

### Permutation testing and an exploratory sample-size calculation provide complementary context

The permutation test did not provide evidence that the observed classification performance exceeded the matched null distribution (p = 0.327). An exploratory sample-size calculation adds useful context: converting the observed cross-validated AUC to an approximate effect size and applying a standard two-sample power formula suggested that detecting an effect of this magnitude with conventional 80% power would require roughly 159 participants, against the 76 available here. We offer this as an approximation, not a firm conclusion; cross-validated AUC values are not independent across folds and repeats, and the calculation cannot on its own distinguish “a real effect exists but this cohort was underpowered to confirm it” from “no meaningful effect exists at all.” What it does provide is a specific, actionable number for the field: future network-based transcriptomic studies, in ADHD and elsewhere, would do well to report this kind of sample-size calculation routinely alongside permutation testing, rather than relying on an unquantified acknowledgment that a larger cohort would help.

### Attention-derived feature importance requires independent validation

The negative correlation between attention weights and perturbation-based importance measures is this study’s most consequential finding, and one that extends well beyond ADHD or this particular dataset. Simple disagreement among the three feature-attribution methods would not, by itself, have been surprising --- three approaches applied to a modestly sized dataset often diverge to some degree. What we found was more specific: attention-derived gene rankings were systematically, inversely related to alternative attribution measures designed to quantify predictive dependence, with a moderate effect size (ρ up to −0.57) that reached high statistical significance despite this cohort’s constraints (S2 Table). The pattern echoes a concern first raised in natural language processing, where attention weights have been shown not to reliably track which inputs a model’s output actually depends on [11], though, to our knowledge, it has rarely been demonstrated this directly in a graph-based model applied to genomic data. A plausible mechanistic account emerges when this finding is considered alongside another: several of the most attention-heavy genes showed broad, non-specific correlation with multiple unrelated blood cell-type marker panels (Fig 8). Attention may have been tracking a dominant technical or structural axis of variation, possibly overall RNA yield or graph connectivity, rather than genes with genuine relevance to the classification task. Whatever the precise mechanism, the practical implication is straightforward: attention weights from a GAT should not, on their own, be treated as evidence of which genes matter biologically (Fig 4; see S1 Fig for a direct before-and-after comparison showing how substantially this pipeline’s gene-level and ablation-based conclusions changed once leakage was corrected, despite comparatively modest change in overall classification performance). Future work that relies on attention-derived rankings as candidate findings would do well to validate those rankings against at least one alternative, perturbation-based method before treating them as more than a starting hypothesis.

### ADHD heterogeneity may obscure reproducible transcriptomic signatures

Five converging null results, across attention, permutation importance, SHAP, and two differential-expression designs, support the same conclusion: no reproducible gene-level ADHD signal was detectable in this cohort. This observation is also consistent with broader evidence that peripheral blood molecular alterations in ADHD are generally subtle and heterogeneous, with systematic reviews reporting considerable variability across studies and biomarkers [16]. Among these, the twin-pair analysis carries particular weight. Discordant monozygotic twin pairs offer close to the strongest genetic control available in observational human research, though with only 16 pairs, the design has limited statistical power of its own. Its null result is therefore best read as valuable orthogonal evidence rather than a more sensitive test than the others reported here, but that it found no signal despite such tight genetic control is a meaningful finding in its own right, not merely one more null result among several.

Recent large-scale genetic studies offer a compelling explanation for these findings [17,18]. That work found that childhood-onset, persistent, and late-diagnosed ADHD are not the same underlying condition observed at different ages: the three phenotypes showed distinct patterns of genetic overlap with intelligence, including 184 shared loci for the combined ADHD phenotype (64 of them novel), yet none detected specifically for persistent ADHD. Functional annotation diverged just as sharply, with childhood-ADHD loci enriched for early neurodevelopmental processes and late-diagnosed loci enriched instead for synapse-related and neuronal signaling pathways. If ADHD subtypes differ this much, down to which biological pathways are implicated, then a cohort that pools them without stratification, as ours did, is not simply a noisier version of one condition. It may be a mixture of conditions whose molecular signatures partially cancel out when averaged together. That offers a plausible, mechanistically specific reason why our pathway-level enrichment analysis (Fig 6) found nothing convergent across methods, beyond the more general explanation of limited power. It also points to a concrete implication for future study design: cohorts stratified by ADHD subtype, even if individually smaller, might be more likely to reveal subtype-specific transcriptomic signal than a larger but heterogeneous cohort like this one.

### Quality-control analyses support the validity of the analytical pipeline

A skeptical reader, faced with this many null findings, might reasonably wonder whether the data-processing or graph-construction pipeline was simply broken. Two observations argue against that: the recovery of two canonical, independently defined blood co-expression modules [7] (Fig 4), and the absence of gross cell-composition confounding at the group level (Fig 8). The limitations of this study are limitations of statistical power and cohort composition, not of implementation, and that distinction should shape how much weight readers place on the negative findings reported here.

Taken together, the value of this work lies less in any single result than in the validation framework itself: statistical significance testing, power analysis, multi-method interpretability comparison, orthogonal differential expression, pathway-level analysis, and data-quality controls, a set of checks that any graph-based transcriptomic study could apply before taking its biological claims at face value. That framework generalizes well beyond ADHD and beyond this dataset. As graph neural networks are increasingly adopted across computational biology and complex disease research, where biological network topology is increasingly incorporated into predictive models [19], rigorous validation of biological interpretation becomes increasingly important. Our findings should not be read as evidence against graph-based models, or against the existence of ADHD-associated transcriptomic alterations. They show instead that rigorous validation, not predictive performance alone, is what justifies interpreting a finding as biologically meaningful, especially in small, heterogeneous cohorts.

### Limitations

Several limitations warrant explicit acknowledgment, in approximate order of importance. First, for both graph-based models, the epoch retained per fold was selected by validation AUC on that same fold’s held-out partition, rather than through a separate inner validation split reserved exclusively for epoch selection or a nested cross-validation design. Because the reported fold AUC is effectively the maximum achieved across 150 correlated evaluations on a small (approximately 15-participant) validation set, this procedure can optimistically bias the resulting AUC estimates upward, and should be regarded as a remaining, unresolved source of potential leakage distinct from the sample-relatedness and feature-selection leakage corrected elsewhere in this study. This procedure was applied identically to the observed and null computations in the permutation test (Methods), which plausibly limits its impact on that specific comparison and may help explain why the null distribution’s mean (0.613) sat well above chance-level (0.5) rather than near it; the same symmetry does not apply to the single-split and repeated cross-validation AUC estimates reported elsewhere, which should be interpreted with this caveat in mind. A full correction (a nested cross-validation redesign, re-run across every analysis in this study) was beyond the scope of the present revision and is an important direction for follow-up work. Second, the modest sample size, even after accounting for the twin sub-cohort’s genetic-control advantages, limited statistical power throughout this study. Third, this analysis draws on a single dataset; a directly comparable, publicly available whole-blood ADHD transcriptomic cohort of adequate size for external replication was not available at the time of this study, though at least one substantially larger peripheral blood mononuclear cell cohort exists in the literature [20] and could offer a valuable, if not perfectly tissue-matched, validation opportunity for future work. Fourth, our graph construction relied on fixed thresholds for co-expression strength and STRING confidence; while consistent with common practice, a systematic sensitivity analysis across alternative thresholds was not conducted, and different choices might alter the specific genes and edges prioritized, though this is unlikely to alter the study’s central conclusions regarding attention-based interpretability. Fifth, our blood cell-type analysis used proxy marker-expression scores from a small, fixed panel of genes per cell type rather than a validated, reference-based deconvolution algorithm; while sufficient to detect gross group-level differences in these marker scores, a more sophisticated approach might reveal subtler compositional confounding not captured here. Sixth, this cohort was not stratified by ADHD subtype or clinical presentation, a limitation discussed above in connection with known subtype-specific genetic heterogeneity. Finally, as a peripheral blood study, any transcriptomic signal detected, had one reached significance, would necessarily reflect systemic or immune-related biology rather than direct neural mechanisms, and inferences about central nervous system processes from peripheral blood findings should always be made cautiously regardless of a study’s statistical outcome.

## Conclusion

Predictive performance and biological interpretability are not the same achievement, and a model that performs reasonably well at the former does not automatically earn the latter. In this study, graph-based models showed modest predictive gains, although these were not confirmed against the permutation-derived null, while an extensive, multi-method validation effort, spanning permutation testing, power analysis, three complementary feature-attribution approaches, two differential expression designs, pathway-level enrichment analysis, and dedicated quality-control checks, found no reproducible gene-level signal, and found that attention-derived rankings were inversely associated with the examined alternative attribution measures and therefore did not provide a reliable guide to feature importance in this analysis. We think this outcome is worth reporting plainly rather than downplaying, because the alternative --- treating attention-derived gene rankings as biological findings without this kind of validation --- is a pattern we suspect is not unique to this study or this disorder. As graph-based models see continued use in genomics and psychiatric research, robust validation methodology deserves to be treated as seriously as predictive accuracy when translating these models into biological claims, particularly in the small-cohort settings that remain common throughout this field.

## Supporting information

S1 Fig Effect of Analytical Pipeline Correction on Gene-Level Attention and Ablation Findings. Comparison of attention-derived gene interaction networks (panels A-B) and node ablation results (panels C-D) computed before versus after correcting sample-relatedness leakage, feature-selection leakage, and transcript-to-gene mapping errors in the analysis pipeline, using the same underlying dataset and graph-attention framework, revised analysis incorporating group-aware cross-validation designed to address sample-relatedness leakage, transcript-to-gene aggregation, and the other methodological corrections described in the revised Methods. Before correction (panels A, C), the literature-derived candidate genes (*IFITM3*, *RSAD2*, *ITGA2B*, *TNS1*, *TRIM58*) appear densely interconnected with high fold robustness (4–5/5) and show uniformly strong, negative AUC changes upon removal, consistent with the original manuscript’s claim that these genes were important drivers of model predictions. After correction (panels B, D), an almost entirely different set of genes emerges as attention-robust, *RSAD2* is the only original candidate gene still present and appears with low robustness (2/5) and a single connection, and node ablation shows two of the original candidate genes (*IFI44L*, *RSAD2*) producing a net *increase* in AUC upon removal, the opposite of the pre-correction pattern. This figure is provided to illustrate concretely, rather than only describe, how substantially leakage correction altered gene-level conclusions in this analysis. This comparison illustrates that rigorous correction of methodological biases can substantially alter biological interpretation while having only a comparatively modest effect on overall predictive performance.
(PNG)

S1 Table Model hyperparameters, random seeds, and software versions. (DOCX)

S2 Table Spearman rank correlation between attention, permutation importance, and SHAP gene rankings. (DOCX)
