# Supplementary material for: Rigorous validation of graph-based network analysis reappraises biologically coherent ADHD-associated transcriptomic modules in peripheral blood
Source: PLoS One. 2026 Oct 5;21(10):e0359656. doi: 10.1371/journal.pone.0359656 (PMC13637919; doi:10.1371/journal.pone.0359656)
Supplement: Supplementary file 1 [file pone.0359656.s002.docx]

**S1 Table. Model hyperparameters, random seeds, and software versions.**

*Graph attention network (GAT)*

- Architecture: two graph attention layers (layer 1: 2 heads, concatenated, 1 → 64 dimensions; layer 2: 1 head, averaged, 64 → 32 dimensions), ELU activation, dropout p = 0.4; global mean pooling; classification head 32 → 16 → 2 (ReLU, dropout)
- Edge features: co-expression correlation strength and STRING confidence score, incorporated as edge attributes at both attention layers
- Optimizer: Adam, learning rate 0.001, L2 weight decay 1×10⁻⁴
- Training: 150 epochs per fold (main cross-validation and repeated cross-validation); 50- and 100-epoch budgets used for the matched observed/null permutation test; 100 epochs for node ablation retraining
- Primary random seed: 1 (main five-fold cross-validation); seeds 0-19 used for the 20-repeat paired cross-validation; permutation-specific seeds used for label shuffling and model initialization in the permutation test

*Graph convolutional network (GCN)*

- Architecture: two graph convolutional layers matching GAT's hidden dimensionality, ELU activation, dropout p = 0.4; global mean pooling; classification head matching GAT
- Edge features: same co-expression and STRING edge weights as GAT, incorporated via weighted graph convolution
- Optimizer, training schedule, and seeds: identical to GAT, for direct comparability

*Classical machine-learning baselines*

- Logistic regression: L2 penalty, default scikit-learn regularization strength, max iterations 1000
- Support vector machine: RBF kernel, default scikit-learn regularization (C = 1.0), probability estimates enabled
- Random forest: 200 estimators, default scikit-learn splitting criteria
- Gradient boosting: 100 estimators, default scikit-learn learning rate and tree depth
- XGBoost: 100 estimators, default XGBoost learning rate and tree depth
- All baselines evaluated under the identical group-stratified, fold-wise feature-selection cross-validation protocol as the graph-based models, using flat (non-graph) expression vectors as input

*Fold structure*

- Five-fold, group-stratified cross-validation throughout, with twin pairs treated as indivisible groups
- 20 independent fold assignments generated for the repeated cross-validation analysis (distinct random seeds per assignment)

*Software environment*

- Python (graph neural network models: PyTorch, PyTorch Geometric; classical baselines and cross-validation utilities: scikit-learn; feature attribution: SHAP; pathway enrichment: gseapy; statistical testing and power analysis: scipy, statsmodels)
- Exact package versions (analysis environment): Python 3.12.13; PyTorch 2.11.0+cu128; PyTorch Geometric 2.8.0.post1; scikit-learn 1.6.1; XGBoost 3.3.0; SHAP 0.52.0; gseapy 1.3.1
