# Supplementary material for: Rigorous validation of graph-based network analysis reappraises biologically coherent ADHD-associated transcriptomic modules in peripheral blood
Source: PLoS One. 2026 Oct 5;21(10):e0359656. doi: 10.1371/journal.pone.0359656 (PMC13637919; doi:10.1371/journal.pone.0359656)
Supplement: Supplementary file 2 [file pone.0359656.s003.docx]

**S2 Table. Spearman rank correlation between attention, permutation importance, and SHAP gene rankings.**

| **Gene set** | **Comparison** | **Spearman ρ** | **p-value** |
| --- | --- | --- | --- |
| Full common set (n = 475) | Attention vs. Permutation importance | −0.353 | < 0.0001 |
| Full common set (n = 475) | Attention vs. SHAP | −0.216 | < 0.0001 |
| Full common set (n = 475) | Permutation importance vs. SHAP | +0.265 | < 0.0001 |
| Top 100 genes | Attention vs. Permutation importance | −0.574 | < 0.0001 |
| Top 100 genes | Attention vs. SHAP | −0.411 | < 0.0001 |
| Top 100 genes | Permutation importance vs. SHAP | +0.117 | 0.0667 |
| Top 200 genes | Attention vs. Permutation importance | −0.509 | < 0.0001 |
| Top 200 genes | Attention vs. SHAP | −0.345 | < 0.0001 |
| Top 200 genes | Permutation importance vs. SHAP | +0.179 | 0.0003 |
| Top 500 genes | Identical to full common set (only 475 genes were scored by all three methods, fewer than 500) | — | — |
