# Supplementary material for: Rigorous validation of graph-based network analysis reappraises biologically coherent ADHD-associated transcriptomic modules in peripheral blood
Source: PLoS One. 2026 Oct 5;21(10):e0359656. doi: 10.1371/journal.pone.0359656 (PMC13637919; doi:10.1371/journal.pone.0359656)
Supplement: Supplementary file 3 [file pone.0359656.s005.docx]

Response to Reviewers

*Manuscript: Rigorous validation of graph-based network analysis reappraises biologically coherent ADHD-associated transcriptomic modules in peripheral blood*

The reviewers' comments prompted a comprehensive re-evaluation of both our analytical pipeline and the interpretation of our findings. Rather than limiting the revision to textual clarification, we reanalyzed the complete dataset using a substantially strengthened analytical framework designed to address the methodological concerns raised during peer review, including leakage-resistant group-stratified cross-validation, fold-wise feature selection, repeated cross-validation, rigorously matched permutation testing, complementary explainability analyses (SHAP and permutation importance), expanded quality-control analyses, and a comprehensive reassessment of the biological interpretation.

These additional analyses materially changed several of the study's conclusions. In particular, while the revised analyses confirmed a modest predictive advantage of graph-based models, they no longer supported strong biological interpretation of attention-derived gene rankings or reproducible ADHD-associated transcriptomic modules. Accordingly, the manuscript was substantially rewritten to reflect the revised evidence, shifting its emphasis from biomarker discovery toward rigorous validation of graph-based transcriptomic analyses.

Consequently, the extensive tracked changes primarily reflect new analyses and the corresponding reinterpretation of the findings rather than stylistic rewriting. Importantly, the underlying dataset, research question, study objectives, and overall experimental design remain unchanged. We believe these revisions substantially strengthen the scientific rigor, transparency, and reproducibility of the work and directly address the reviewers' concerns.

To facilitate evaluation of the revised manuscript, we also added Supplementary S1 Fig. , which directly contrasts the principal gene-level findings before and after correction of the analytical pipeline. This figure illustrates why the biological conclusions changed despite only modest changes in predictive performance.

| **Reviewer** | **Comment** | **Response** | **Manuscript Location** |
| --- | --- | --- | --- |
| **Editor** | E1: Manuscript should follow PLOS ONE style templates and file-naming conventions. | The manuscript has been entirely reformatted to comply with current PLOS ONE submission guidelines: double-spaced text, continuous line numbers, page numbers, proper Word heading styles (Heading 1/2, limited to two levels), Vancouver/ICMJE reference style, figure captions inserted inline immediately after first citation, and Competing Interests/Financial Disclosure statements removed from the manuscript file (to be entered via the submission system as required). | Throughout; see in particular manuscript-wide formatting (spacing, line numbers, headings) and References |
| **Editor** | E2: Reviewers should be able to locate a clear reference to Figure 7 in the manuscript text. | Figure 7 is now explicitly cited in the Results section in the paragraph reporting the paired differential-expression analysis of the 16 discordant monozygotic twin pairs. All figures were also renumbered and verified to be cited in ascending numerical order. | Results Section |
| **Editor** | E3: Figure 7 requires a caption. | All figures now have complete captions inserted inline in the manuscript text, immediately following the paragraph in which each is first cited, per current PLOS ONE guidelines. | Results section |
| **Editor** | E4: Consider citing recent work on subtype-dependent genetic architecture in ADHD (PMID 42298576) to help interpret the transcriptomic findings. | This reference (Zhao, Yang, Liu et al., BMC Medicine, 2026) has been incorporated substantively into the Discussion. Its finding that childhood-onset, persistent, and late-diagnosed ADHD show distinct genetic loci and functional pathway enrichment is used to explain why a cohort pooling these subtypes without stratification may show diluted or non-convergent transcriptomic signal, complementing our own null gene-level findings. | Discussion, "ADHD heterogeneity may obscure reproducible transcriptomic signatures"; Reference 10 |
| **Reviewer 1** | R1.1: The manuscript should distinguish the finding of limited linear separability from claims about co-regulatory biology motivating the graph-based approach. | The Introduction and Discussion have been revised to state precisely what the data support: graph-based models modestly outperform classical machine-learning approaches, which is presented as evidence that network topology adds predictive information, not as confirmation of any specific co-regulatory biological mechanism. | Introduction, paragraph 2; Discussion, "Graph-based models provide modest predictive benefit beyond conventional machine learning" |
| **Reviewer 1** | R1.2: Language throughout the manuscript should be softened where it implies established biological findings. | The manuscript has been substantially rewritten throughout to frame attention-derived findings as hypothesis-generating rather than mechanistic evidence, consistent with the paper's overall reframing as a validation-focused study rather than a biomarker-discovery study. | Discussion and Conclusion, throughout |
| **Reviewer 1** | R1.3: Twin-pair samples may be split across training and validation folds within cross-validation, risking leakage. | Addressed. Group-stratified five-fold cross-validation now treats each of the 16 discordant monozygotic twin pairs as a single, indivisible group, guaranteeing both twins in a pair always fall on the same side of the training/validation boundary. This constraint is verified programmatically for every fold generated in the study (Methods, "Leakage-resistant cross-validation design"). | Methods, "Leakage-resistant cross-validation design" |
| **Reviewer 1** | R1.4: Gene selection appears to have been performed prior to the cross-validation split, risking feature-selection leakage. | Addressed. Gene selection (top 300 most variable genes) is now performed independently within each cross-validation fold, using only that fold's training participants, never once across the entire dataset prior to splitting (Methods, "Preprocessing and gene selection"; illustrated in new Fig 1). | Methods, "Preprocessing and gene selection" |
| **Reviewer 1** | R1.5: The term "sex-adjusted gene selection" requires clarification. | Clarified in Methods: a fixed panel of Y-chromosome-encoded genes is excluded prior to variance ranking to reduce sex confounding in feature selection specifically. The manuscript now explicitly states this does not substitute for a fully sex-stratified analysis, which remains a limitation given the unavailability of individual-level sex metadata in the public dataset. | Methods, "Preprocessing and gene selection", paragraph 2 (Y-chromosome gene exclusion) |
| **Reviewer 1** | R1.6: Hyperparameter tuning procedures should be documented consistently, ideally in a supplementary table. | Addressed. A new S1 Table reports complete model architectures, hyperparameters, random seeds, and software versions for both graph-based models and all five classical baselines. | S1 Table (Supporting Information); referenced in Methods, "GAT and GCN architecture and training" |
| **Reviewer 1** | R1.7: Attention weights combined with single-gene ablation are insufficient to establish biological importance; additional validation methods are needed. | Addressed and substantially extended. Two additional, independent feature-importance methods were implemented: permutation importance and SHAP. Comparing all three methods revealed that attention-derived gene rankings are significantly negatively correlated with both alternative measures (Spearman rho up to -0.57, p<0.0001), a stronger and more specific finding than mere non-convergence, and now represents one of the principal methodological findings of the revised manuscript. (Results, "Attention-derived gene rankings were negatively correlated with perturbation-based importance measures"; Discussion). | Methods, "Feature robustness: attention weights, permutation importance, and SHAP"; Results, "Attention-derived gene rankings were negatively correlated with perturbation-based importance measures"; Discussion, "Attention-derived feature importance requires independent validation" |
| **Reviewer 1** | R1.8: The Limitations section should more fully discuss sample size, absence of a replication cohort, and potential confounds. | The Limitations section has been substantially expanded to seven explicit points, ordered by importance: an epoch-selection procedure newly identified as a potential residual source of leakage, sample size and statistical power (quantified via an exploratory sample-size calculation), single-dataset generalizability (with a candidate replication cohort identified in the literature), graph-construction threshold sensitivity, the simplified cell-type marker approach, lack of ADHD subtype stratification, and the peripheral-blood-versus-brain interpretive caveat. | Discussion, "Limitations" (all seven numbered points) |
| **Reviewer 1** | R1.9: The GWAS/eQTL convergence analysis and MDD cross-disorder comparison should be presented as exploratory rather than confirmatory. | Both sections have been removed from the manuscript entirely. The corrected analysis pipeline no longer identifies the original candidate gene list on which these sections were built, so presenting them even as "exploratory" would risk implying a level of support the corrected data do not provide. | *Section removed entirely (previously present in the original submission; not part of the revised manuscript)* |
| **Reviewer 1** | R1.10 Provide a workflow figure and a detailed Supplementary Methods section showing the exact sequence. | The entire figure set was restructured as part of this revision. A new Fig 1 (analysis pipeline workflow diagram) was added to address exactly this kind of clarity concern, and all figures (now Fig 1-8) were renumbered and verified to be cited in strict ascending numerical order at first appearance, per PLOS ONE requirements. | Fig 1 (Methods, "Analysis pipeline overview"); Methods Section. |
| **Reviewer 2** | R2.1: The sample size is small, and fold-level AUC varies substantially (0.500-0.839); repeated cross-validation with bootstrap confidence intervals is needed. | Addressed. A repeated cross-validation analysis (20 independent group-stratified splits) was added, with paired comparison of GAT and GCN mean AUC and 95% confidence intervals (Methods, "Repeated cross-validation and paired GAT-GCN comparison"; Results). A rigorously matched permutation test and an exploratory sample-size calculation were also added to directly contextualize the effect of limited sample size. | Methods, "Repeated cross-validation and paired GAT-GCN comparison" and "Permutation test"; Results, "Graph-based models modestly outperformed classical machine-learning baselines" and "Performance did not exceed a permutation-based null..." |
| **Reviewer 2** | R2.2: Findings are based on a single dataset; the exploratory nature of the study should be acknowledged, and identified genes should be checked against previously published ADHD transcriptomic studies. | The exploratory, hypothesis-generating framing is now adopted throughout the manuscript, and a substantially larger PBMC-based ADHD transcriptomic cohort (Mortimer et al., 2020) is identified in the Limitations as a candidate external validation dataset for future work. A direct, gene-by-gene comparison against previously published ADHD transcriptomic candidate gene lists was not performed in this revision and is noted as an item for future work rather than claimed as complete. | Discussion, "Limitations", point 3 (single-dataset generalizability); throughout, hypothesis-generating framing |
| **Reviewer 2** | R2.3: Graph construction parameters (co-expression correlation threshold, STRING confidence threshold) require justification or a sensitivity analysis. | We appreciate this suggestion. Although a systematic parameter sensitivity analysis was beyond the scope of the present revision, we have expanded the Methods to justify the selected thresholds, which are consistent with common practice, and now explicitly acknowledge this as a limitation and an important direction for future work. | Discussion, "Limitations", point 4 |
| **Reviewer 2** | R2.4: Attention weights alone are not sufficient evidence of biological importance; validation against permutation importance or SHAP is needed. | Addressed and substantially extended, as for R1.7 above. This comment's underlying concern is now the manuscript's central finding: attention-derived rankings are significantly negatively correlated with both permutation importance and SHAP, indicating attention should not be treated as a reliable guide to feature importance in this setting. | Methods, "Feature robustness..."; Results, "Attention-derived gene rankings were negatively correlated..."; Discussion, "Attention-derived feature importance requires independent validation" |
| **Reviewer 2** | R2.5: Blood is not brain; claims regarding neuroinflammation or CNS mechanisms should be moderated. | Addressed. Given the corrected pipeline's null gene-level findings, claims regarding specific neuroinflammatory or CNS mechanisms have been removed. The manuscript now explicitly states that any peripheral blood transcriptomic signal, had one reached significance, would necessarily reflect systemic or immune-related biology rather than direct neural mechanisms, and that inferences about CNS processes from peripheral blood findings should always be made cautiously (Limitations, final point). | Discussion, "Limitations", final point |
| **Reviewer 2** | R2.6: GWAS/eQTL enrichment claims require formal statistical testing rather than descriptive overlap. | Addressed by removing the section. As for R1.9, the GWAS/eQTL convergence analysis has been removed from the manuscript entirely, since the underlying candidate gene list is no longer supported by the corrected pipeline; presenting even a formally-tested version of this analysis would not be a meaningful addition given that context. | *Section removed entirely (previously present in the original submission; not part of the revised manuscript)* |
| **Reviewer 2** | R2.7: Terminology such as "ADHD-associated" should be used more cautiously throughout. | Addressed. Cautious, hedged language is now used consistently throughout the manuscript (e.g., "hypothesis-generating," "modest," "reappraises" in the title itself), and the framing throughout is explicit that predictive performance and biological interpretation are treated as distinct questions. | Throughout, particularly Abstract, Discussion, and Conclusion |
| **Reviewer 2** | R2.8: The MDD cross-disorder comparison has platform and methodological confounds that need to be addressed. | Addressed by removing the section. As for R1.9, the MDD cross-disorder comparison has been removed from the manuscript entirely, since it was built to demonstrate specificity of a gene signature that the corrected pipeline no longer supports. | *Section removed entirely (previously present in the original submission; not part of the revised manuscript)* |
| **Reviewer 2** | R2.9: Machine-learning procedure reporting (hyperparameters, random seeds, software versions) requires more detail. | Addressed. A new S1 Table provides complete architecture, hyperparameter, random seed, and software version details for all graph-based and classical models. | S1 Table (Supporting Information) |
