# Supplementary material for: Rigorous validation of graph-based network analysis reappraises biologically coherent ADHD-associated transcriptomic modules in peripheral blood
Source: PLoS One. 2026 Oct 5;21(10):e0359656. doi: 10.1371/journal.pone.0359656 (PMC13637919; doi:10.1371/journal.pone.0359656)
Supplement: Supplementary file 4 [file pone.0359656.s006.docx]

Response to Reviewers

*Manuscript:* ***Rigorous validation of graph-based network analysis reappraises biologically coherent ADHD-associated transcriptomic modules in peripheral blood***

We thank the reviewers for their careful re-evaluation of the revised manuscript. Reviewer 1 indicated that all previous comments had been addressed and had no further comments. Reviewer 2 considered the manuscript technically sound and raised two remaining concerns regarding the characterization of residual leakage and the interpretation of the graph-based predictive findings. We addressed both points through targeted revisions to the manuscript’s terminology and interpretation, without introducing additional analyses. The revised manuscript now clearly distinguishes the sources of leakage that were explicitly corrected from the remaining epoch-selection limitation, and it consistently presents the predictive findings as modest and statistically uncertain rather than as evidence of a definitive graph-based advantage. These revisions preserve the study’s results while aligning the interpretation more closely with the statistical evidence.

| **Reviewer** | **Comment** | **Response** | **Manuscript Location** |
| --- | --- | --- | --- |
| **Reviewer 1** | All comments have been addressed; no further comments. | We thank Reviewer 1 for the positive reassessment of the revised manuscript and for confirming that the previous concerns were fully addressed. No additional changes were required in response to Reviewer 1. | No additional manuscript changes required. |
| **Reviewer 2** | **R2.1**: The term “leakage-resistant” is too strong because the manuscript acknowledges a remaining source of potential model-selection leakage/optimistic bias: the best epoch is selected using the same held-out validation partition on which fold performance is reported. | Addressed. We removed the term “leakage-resistant” and replaced it with more precise language describing exactly which sources of leakage were addressed. In the Abstract, the pipeline is now described as “leakage-aware.” The Methods heading was revised to “Cross-validation design addressing sample-relatedness and feature-selection leakage,” and corresponding wording throughout the manuscript now refers specifically to the corrected sample-relatedness and feature-selection issues rather than implying complete immunity to leakage. Importantly, the Limitations section continues to state explicitly that selecting the best epoch by validation AUC on the same held-out fold may optimistically bias AUC estimates and remains an unresolved source of potential leakage; a fully nested cross-validation redesign is identified as future work. This change directly distinguishes corrected leakage sources from the residual epoch-selection limitation. | Abstract, Methods (“Cross-validation design addressing sample-relatedness and feature-selection leakage”), Discussion/Limitations (first limitation), and Supporting Information S1 Fig caption. |
| **Reviewer 2** | **R2.2**: Claims of a graph-based predictive advantage should be toned down because the GAT–GCN difference is modest (mean AUC 0.624 vs. 0.599; paired p = 0.049), overall GAT performance was not statistically distinguishable from the matched permutation-derived null (p = 0.327), and some optimistic bias may remain. | Addressed. We revised the Abstract, Results, Discussion, and Conclusion to present the predictive findings more cautiously and to separate the small GAT–GCN difference from the permutation-test result. The Abstract now states that GAT achieved a “slightly higher mean AUC” than GCN and immediately notes that GAT performance was not statistically distinguishable from the matched permutation-derived null. The Results heading now reads “Graph-based models showed modest predictive gains over classical machine-learning baselines,” and the repeated GAT–GCN result is described as a “small and statistically fragile difference.” The Discussion similarly states that repeated cross-validation showed only a small GAT–GCN difference, while the permutation section now explicitly states that the test “did not provide evidence that the observed classification performance exceeded the matched null distribution.” The Conclusion was also moderated to state that graph-based models showed modest predictive gains that were not confirmed against the permutation-derived null. Together, these revisions avoid presenting the observed performance differences as definitive evidence of a graph-based predictive advantage. | Abstract (Results and Conclusions); Introduction (study questions); Results (“Graph-based models showed modest predictive gains…” and “Performance did not exceed a permutation-based null…”); Discussion (opening, model-comparison subsection, permutation subsection); Conclusion. |

We appreciate Reviewer 2’s comments, which helped us make the manuscript’s terminology and interpretation more precise. We believe the revised wording now fully reflects the scope of the corrected methodological issues and the statistical uncertainty of the predictive findings.
