# Supplementary material for: Rigorous validation of graph-based network analysis reappraises biologically coherent ADHD-associated transcriptomic modules in peripheral blood
Source: PLoS One. 2026 Oct 5;21(10):e0359656. doi: 10.1371/journal.pone.0359656 (PMC13637919; doi:10.1371/journal.pone.0359656)
Supplement: Peer review history

# Peer review: Decision Letter 0

**DOI:** 10.1371/journal.pone.0359656.r001

**Authors:** Feng Liu

20 Jul 2026

Dear Dr. Ali,

Thank you for submitting your manuscript to PLOS ONE. After careful consideration, we feel that it has merit but does not fully meet PLOS ONE’s publication criteria as it currently stands. Therefore, we invite you to submit a revised version of the manuscript that addresses the points raised during the review process.

Please submit your revised manuscript by Sep 18 2026 11:59PM. If you will need more time than this to complete your revisions, please reply to this message or contact the journal office at plosone@plos.org. When you're ready to submit your revision, log on to https://www.editorialmanager.com/pone/ and select the 'Submissions Needing Revision' folder to locate your manuscript file.

- A letter that responds to each point raised by the academic editor and reviewer(s). You should upload this letter as a separate file labeled 'Response to Reviewers'.
- A marked-up copy of your manuscript that highlights changes made to the original version. You should upload this as a separate file labeled 'Revised Manuscript with Track Changes'.
- An unmarked version of your revised paper without tracked changes. You should upload this as a separate file labeled 'Manuscript'.

If you would like to make changes to your financial disclosure, please include your updated statement in your cover letter. Guidelines for resubmitting your figure files are available below the reviewer comments at the end of this letter.

If applicable, we recommend that you deposit your laboratory protocols in protocols.io to enhance the reproducibility of your results. Protocols.io assigns your protocol its own identifier (DOI) so that it can be cited independently in the future. For instructions see: https://journals.plos.org/plosone/s/submission-guidelines#loc-laboratory-protocols. Additionally, PLOS ONE offers an option for publishing peer-reviewed Lab Protocol articles, which describe protocols hosted on protocols.io. Read more information on sharing protocols at https://plos.org/protocols?utm_medium=editorial-email&utm_source=authorletters&utm_campaign=protocols.

As the corresponding author, your ORCID iD is verified in the submission system and will appear in the published article. PLOS supports the use of ORCID, and we encourage all coauthors to register for an ORCID iD and use it as well. Please encourage your coauthors to verify their ORCID iD within the submission system before final acceptance, as unverified ORCID iDs will not appear in the published article. *Only*  the individual author can complete the verification step; PLOS staff *cannot*  verify ORCID iDs on behalf of authors.

We look forward to receiving your revised manuscript.

Kind regards,

Feng Liu, Ph.D.

Academic Editor

PLOS One

Journal Requirements:

When submitting your revision, we need you to address these additional requirements.

1. Please ensure that your manuscript meets PLOS ONE's style requirements, including those for file naming. The PLOS ONE style templates can be found at

https://journals.plos.org/plosone/s/file?id=wjVg/PLOSOne_formatting_sample_main_body.pdf and https://journals.plos.org/plosone/s/file?id=ba62/PLOSOne_formatting_sample_title_authors_affiliations.pdf

2. Please ensure that you refer to Figure 7 in your text as, if accepted, production will need this reference to link the reader to the figure.

3. Please include a caption for figure 7.

4. If the reviewer comments include a recommendation to cite specific previously published works, please review and evaluate these publications to determine whether they are relevant and should be cited. There is no requirement to cite these works unless the editor has indicated otherwise.

Additional Editor Comments :

I have now received and carefully considered the comments from two independent reviewers. Both reviewers found the topic of this manuscript interesting and recognized the potential value of applying graph-based approaches to investigate transcriptomic patterns associated with ADHD. However, both reviewers raised several important concerns that should be addressed before the manuscript can be further considered for publication. Please carefully consider and respond to all reviewer comments during revision.

In addition to the reviewer comments, I would like to highlight the following point for further consideration:

The authors may consider expanding the discussion regarding ADHD heterogeneity and its implications for molecular investigations. Recent genetic studies have demonstrated that ADHD subtypes may exhibit partially distinct biological architectures, suggesting that molecular signatures associated with ADHD may also vary across clinical presentations. Relevant recent work on subtype-dependent genetic architecture in ADHD (e.g., PMID: 42298576) may provide useful context for interpreting the transcriptomic findings reported in this study.

[Note: HTML markup is below. Please do not edit.]

Reviewers' comments:

Reviewer's Responses to Questions

**Comments to the Author**

1. Is the manuscript technically sound, and do the data support the conclusions?

Reviewer #1: Yes

Reviewer #2: Partly

**********

2. Has the statistical analysis been performed appropriately and rigorously?

Reviewer #1: Yes

Reviewer #2: Yes

**********

3. Have the authors made all data underlying the findings in their manuscript fully available?

The PLOS Data policy

Reviewer #1: Yes

Reviewer #2: Yes

**********

4. Is the manuscript presented in an intelligible fashion and written in standard English?

Reviewer #1: Yes

Reviewer #2: Yes

**********

Reviewer #1: This study applies a graph attention network framework to pediatric ADHD whole-blood RNA-seq data and identifies candidate co-expression modules related to interferon/immune signaling and platelet/cell adhesion. This is overall a nice work, and I hope my following comments can help improve the presentation of the manuscript.

Please distinguish more clearly between limited linear separability, the motivation for graph-based modelling, and the biological interpretation of model-prioritized genes. Does poor performance of a linear model necessarily imply co-regulatory biology, or does it simply justify testing alternative modelling strategies? This distinction should be made explicit.

The current wording occasionally reads as if the identified modules represent established ADHD-associated biology.

The dataset includes a discordant monozygotic twin sub-cohort and an independent case-control sub-cohort. Were twin pairs kept within the same fold during the combined-cohort five-fold cross-validation? Please clarify this in the Methods and provide a group-aware cross-validation sensitivity analysis in the Supplementary Materials.

Was variance-based gene selection performed within each training fold, or was the 300-gene feature set selected before cross-validation? Please provide a workflow figure and a detailed Supplementary Methods section showing the exact sequence.

The term “sex-adjusted gene selection” seems too strong. Excluding Y-chromosome genes is not equivalent to adjustment for sex-related transcriptomic variation.

Were hyperparameters tuned comparably across GAT, GCN, XGBoost, random forest, SVM, gradient boosting, and logistic regression? Were the same fold assignments used throughout? Please add a supplementary table reporting model specifications, tuning grids or fixed hyperparameters, training settings, seeds, software versions, and fold-level results.

Attention weights and single-gene ablation do not establish independent biological importance. Please consider adding module-level ablation, permutation-based feature importance, or another attribution sensitivity analysis.

The current manuscript acknowledges some constraints, but it does not yet fully explain how these constraints should shape interpretation of the findings. How robust are the results given the small sample size and the lack of an independent ADHD replication cohort? To what extent could residual sex confounding, blood cell composition, limited clinical metadata, or the structure of the twin sub-cohort influence the identified modules? The GWAS/eQTL convergence and the MDD comparison should also be presented as exploratory contextual analyses rather than confirmatory evidence, particularly given the differences in cohort design and sequencing platform.

Reviewer #2: This manuscript presents an interesting computational framework using a GAT to investigate ADHD-associated transcriptomic patterns in peripheral blood. The authors address an important question regarding whether disease-related molecular signals may be encoded in gene co-regulatory structures rather than individual gene expression differences. The integration of graph neural networks with transcriptomic data, together with GWAS and eQTL-based analyses, represents an innovative and potentially valuable approach. The manuscript also demonstrates methodological awareness by implementing leakage-free graph construction and comparing the proposed model with multiple baseline approaches. However, several important issues need to be addressed before the conclusions can be fully supported.

Major Comments:

1. The major concern is the relatively small sample size used for developing and evaluating the deep learning model. The study includes only 76 individuals (39 ADHD and 37 controls), whereas graph neural networks generally require substantially larger datasets to achieve stable and generalizable performance. Although the authors applied five-fold cross-validation and permutation testing, the reported performance shows considerable variability across folds (AUC ranging from 0.500 to 0.839), suggesting that the model may be sensitive to sample partitioning and potentially affected by overfitting. The authors should provide additional analyses to demonstrate the robustness of the model, such as repeated cross-validation with multiple random splits, bootstrap-derived confidence intervals, and more detailed information regarding model complexity relative to the available sample size. Furthermore, the authors should consider moderating the interpretation of the predictive performance and emphasize that the proposed framework represents an exploratory approach rather than a clinically applicable classification model.

2. The generalizability of the identified transcriptomic modules remains uncertain because all major findings were derived from a single ADHD dataset (GSE159104). Although the authors performed a cross-disorder comparison using an independent MDD dataset, this analysis does not serve as an external validation of ADHD-associated modules. Independent replication is particularly important given the small cohort size and the complexity of the proposed deep learning framework. If additional ADHD transcriptomic datasets are unavailable, the authors should clearly acknowledge that the identified modules represent exploratory, dataset-specific signatures rather than broadly applicable ADHD biomarkers. Moreover, the authors should consider evaluating whether the identified key genes, including IFITM3, IFI44L, RSAD2, ITGA2B, and TNS1, show consistent associations in previously published ADHD transcriptomic studies.

3. The strategy used to construct the gene interaction graph requires further justification. The authors integrated expression-based co-expression networks with STRING protein–protein interaction networks; however, several methodological choices may substantially influence the resulting graph structure and downstream findings. Specifically, the rationale for selecting the top 300 most variable genes as graph nodes, using an absolute Pearson correlation threshold of 0.6 for co-expression edges, and selecting a STRING confidence threshold of 0.4 should be clarified. The authors should provide sensitivity analyses evaluating whether the identified modules remain stable under alternative graph construction strategies, including different numbers of selected genes, alternative correlation thresholds, and different protein interaction confidence thresholds. Demonstrating robustness to these parameter choices would substantially strengthen confidence in the identified biological modules.

4. The interpretation of GAT attention weights as biologically important features requires additional caution. Although attention mechanisms can highlight features contributing to model predictions, attention coefficients do not necessarily represent biological importance or causal relevance. They may reflect model-specific optimization patterns rather than robust biological signals. Therefore, the authors should consider validating the identified genes using additional explainability approaches, such as permutation-based feature importance, integrated gradients, or SHAP analysis. Concordance across multiple interpretation methods would provide stronger evidence that the identified genes represent reproducible biological features rather than artifacts specific to the GAT architecture.

5. The biological interpretation of peripheral blood transcriptomic signatures in relation to ADHD brain mechanisms requires further clarification. The manuscript frequently discusses neuroinflammation, neurodevelopment, and ADHD pathophysiology based on blood-derived expression patterns. However, peripheral blood alterations may reflect systemic immune changes rather than molecular processes directly occurring in the brain. The authors should strengthen the biological relevance of their findings by incorporating additional evidence from brain-related datasets, such as GTEx brain expression, PsychENCODE, or single-cell transcriptomic resources, if available. Alternatively, the authors should moderate statements suggesting direct involvement of these blood-derived signatures in ADHD neural mechanisms and clearly distinguish between peripheral molecular associations and brain-specific biological mechanisms.

6. The integration between transcriptomic modules and ADHD GWAS findings is currently mainly descriptive and requires stronger statistical support. The authors report convergence between GAT-derived modules and ADHD genetic risk genes through pathway overlap; however, it remains unclear whether these overlaps are statistically significant beyond random expectation. The authors should provide enrichment analyses using appropriate background gene sets and consider incorporating more formal statistical genetics approaches, such as MAGMA gene-level association analysis, transcriptome-wide association analysis (TWAS), or colocalization analyses between ADHD genetic risk and expression regulation. These analyses would provide stronger evidence linking the identified transcriptomic modules with ADHD genetic susceptibility.

Minor Comments:

7. The terminology used throughout the manuscript, particularly the phrase “ADHD-associated transcriptomic modules,” may imply that the identified modules represent established ADHD biomarkers. Given that the findings are based on a relatively small single cohort, more cautious terminology may be appropriate. The authors may consider using terms such as “ADHD-related transcriptomic modules” or “ADHD-discriminative blood transcriptomic modules” to better reflect the associative and exploratory nature of the findings.

8. The cross-disorder comparison between ADHD and MDD is potentially interesting but requires more cautious interpretation. The two datasets differ substantially in several aspects, including sequencing platforms, sample sizes, and cohort characteristics. Therefore, differences in transcriptomic signal structure may reflect technical or sampling differences rather than true disorder-specific biological architectures. The authors should further acknowledge these potential confounding factors and avoid overinterpreting the comparison between ADHD and MDD.

9. The manuscript would benefit from more comprehensive reporting of machine learning procedures to facilitate reproducibility. Additional details regarding hyperparameter optimization, whether model tuning was performed exclusively within training folds, early stopping criteria, handling of random initialization, and the complete architecture of the final model should be provided. These details are important for independent replication of the proposed computational framework.

**********

what does this mean?). If published, this will include your full peer review and any attached files.

If you choose “no”, your identity will remain anonymous but your review may still be made public.

**Do you want your identity to be public for this peer review?**  For information about this choice, including consent withdrawal, please see our Privacy Policy

Reviewer #1: No

Reviewer #2: No

**********

[NOTE: If reviewer comments were submitted as an attachment file, they will be attached to this email and accessible via the submission site. Please log into your account, locate the manuscript record, and check for the action link "View Attachments". If this link does not appear, there are no attachment files.]

To ensure your figures meet our technical requirements, please review our figure guidelines: https://journals.plos.org/plosone/s/figures

You may also use PLOS’s free figure tool, NAAS, to help you prepare publication quality figures: https://journals.plos.org/plosone/s/figures#loc-tools-for-figure-preparation.

NAAS will assess whether your figures meet our technical requirements by comparing each figure against our figure specifications.

# Author comment: Author response to Decision Letter 1

**DOI:** 10.1371/journal.pone.0359656.r002

9 Aug 2026

The reviewers' comments prompted a comprehensive re-evaluation of both our analytical pipeline and the interpretation of our findings. Rather than limiting the revision to textual clarification, we reanalyzed the complete dataset using a substantially strengthened analytical framework designed to address the methodological concerns raised during peer review, including leakage-resistant group-stratified cross-validation, fold-wise feature selection, repeated cross-validation, rigorously matched permutation testing, complementary explainability analyses (SHAP and permutation importance), expanded quality-control analyses, and a comprehensive reassessment of the biological interpretation.

These additional analyses materially changed several of the study's conclusions. In particular, while the revised analyses confirmed a modest predictive advantage of graph-based models, they no longer supported strong biological interpretation of attention-derived gene rankings or reproducible ADHD-associated transcriptomic modules. Accordingly, the manuscript was substantially rewritten to reflect the revised evidence, shifting its emphasis from biomarker discovery toward rigorous validation of graph-based transcriptomic analyses.

Consequently, the extensive tracked changes primarily reflect new analyses and the corresponding reinterpretation of the findings rather than stylistic rewriting. Importantly, the underlying dataset, research question, study objectives, and overall experimental design remain unchanged. We believe these revisions substantially strengthen the scientific rigor, transparency, and reproducibility of the work and directly address the reviewers' concerns.

To facilitate evaluation of the revised manuscript, we also added Supplementary S1 Fig. , which directly contrasts the principal gene-level findings before and after correction of the analytical pipeline. This figure illustrates why the biological conclusions changed despite only modest changes in predictive performance.

**All comments of the respected Editor and reviewers were addressed and responded to in the attached "Response_to_Reviewers_Final_05082026.docx" file

Attachment Submitted filename: Response_to_Reviewers_Final_05082026.docx

# Peer review: Decision Letter 1

**DOI:** 10.1371/journal.pone.0359656.r003

**Authors:** Feng Liu

3 Sep 2026

Dear Dr. Ali,

Thank you for submitting your manuscript to PLOS One. After careful consideration, we feel that it has merit but does not fully meet PLOS One’s publication criteria as it currently stands. Therefore, we invite you to submit a revised version of the manuscript that addresses the points raised during the review process.

Please submit your revised manuscript by Oct 18 2026 11:59PM. If you will need more time than this to complete your revisions, please reply to this message or contact the journal office at plosone@plos.org. When you're ready to submit your revision, log on to https://www.editorialmanager.com/pone/ and select the 'Submissions Needing Revision' folder to locate your manuscript file.

- A letter that responds to each point raised by the academic editor and reviewer(s). You should upload this letter as a separate file labeled 'Response to Reviewers'.
- A marked-up copy of your manuscript that highlights changes made to the original version. You should upload this as a separate file labeled 'Revised Manuscript with Track Changes'.
- An unmarked version of your revised paper without tracked changes. You should upload this as a separate file labeled 'Manuscript'.

If you would like to make changes to your financial disclosure, please include your updated statement in your cover letter. Guidelines for resubmitting your figure files are available below the reviewer comments at the end of this letter.

If applicable, we recommend that you deposit your laboratory protocols in protocols.io to enhance the reproducibility of your results. Protocols.io assigns your protocol its own identifier (DOI) so that it can be cited independently in the future. For instructions see: https://journals.plos.org/plosone/s/submission-guidelines#loc-laboratory-protocols. Additionally, PLOS One offers an option for publishing peer-reviewed Lab Protocol articles, which describe protocols hosted on protocols.io. Read more information on sharing protocols at https://plos.org/protocols?utm_medium=editorial-email&utm_source=authorletters&utm_campaign=protocols.

As the corresponding author, your ORCID iD is verified in the submission system and will appear in the published article. PLOS supports the use of ORCID, and we encourage all coauthors to register for an ORCID iD and use it as well. Please encourage your coauthors to verify their ORCID iD within the submission system before final acceptance, as unverified ORCID iDs will not appear in the published article. *Only*  the individual author can complete the verification step; PLOS staff *cannot*  verify ORCID iDs on behalf of authors.

We look forward to receiving your revised manuscript.

Kind regards,

Feng Liu, Ph.D.

Academic Editor

PLOS One

Journal Requirements:

If the reviewer comments include a recommendation to cite specific previously published works, please review and evaluate these publications to determine whether they are relevant and should be cited. There is no requirement to cite these works unless the editor has indicated otherwise.

Please review your reference list to ensure that it is complete and correct. If you have cited papers that have been retracted, please include the rationale for doing so in the manuscript text, or remove these references and replace them with relevant current references. Any changes to the reference list should be mentioned in the rebuttal letter that accompanies your revised manuscript. If you need to cite a retracted article, indicate the article’s retracted status in the References list and also include a citation and full reference for the retraction notice.

[Note: HTML markup is below. Please do not edit.]

Reviewers' comments:

Reviewer's Responses to Questions

**Comments to the Author**

Reviewer #1: All comments have been addressed

Reviewer #2: (No Response)

**********

2. Is the manuscript technically sound, and do the data support the conclusions?

Reviewer #1: Yes

Reviewer #2: Yes

**********

3. Has the statistical analysis been performed appropriately and rigorously?

Reviewer #1: Yes

Reviewer #2: Yes

**********

4. Have the authors made all data underlying the findings in their manuscript fully available?

The PLOS Data policy

Reviewer #1: Yes

Reviewer #2: Yes

**********

5. Is the manuscript presented in an intelligible fashion and written in standard English?

Reviewer #1: Yes

Reviewer #2: Yes

**********

Reviewer #1: The authors reasonably replied to all my previous criticisms and comments. The paper was significantly improved. I have no further comments.

Reviewer #2: I wish to thank the authors for addressing my comments. I have no other comments for review aside from the following two points:

First, the term “leakage-resistant” should be reconsidered, as the authors acknowledge a remaining source of model-selection leakage. More precise wording would be appropriate.

Second, claims regarding the predictive advantage of graph-based models should be further moderated, given the modest GAT–GCN difference, non-significant permutation test, and potential optimistic bias in performance estimation.

**********

what does this mean?). If published, this will include your full peer review and any attached files.

If you choose “no”, your identity will remain anonymous but your review may still be made public.

**Do you want your identity to be public for this peer review?**  For information about this choice, including consent withdrawal, please see our Privacy Policy

Reviewer #1: No

Reviewer #2: No

**********

[NOTE: If reviewer comments were submitted as an attachment file, they will be attached to this email and accessible via the submission site. Please log into your account, locate the manuscript record, and check for the action link "View Attachments". If this link does not appear, there are no attachment files.]

To ensure your figures meet our technical requirements, please review our figure guidelines: https://journals.plos.org/plosone/s/figures

You may also use PLOS’s free figure tool, NAAS, to help you prepare publication quality figures: https://journals.plos.org/plosone/s/figures#loc-tools-for-figure-preparation.

NAAS will assess whether your figures meet our technical requirements by comparing each figure against our figure specifications.

# Author comment: Author response to Decision Letter 2

**DOI:** 10.1371/journal.pone.0359656.r004

4 Sep 2026

Reviewer Comment Response Manuscript Location

Reviewer 1 All comments have been addressed; no further comments. We thank Reviewer 1 for the positive reassessment of the revised manuscript and for confirming that the previous concerns were fully addressed. No additional changes were required in response to Reviewer 1. No additional manuscript changes required.

Reviewer 2 R2.1: The term “leakage-resistant” is too strong because the manuscript acknowledges a remaining source of potential model-selection leakage/optimistic bias: the best epoch is selected using the same held-out validation partition on which fold performance is reported. Addressed. We removed the term “leakage-resistant” and replaced it with more precise language describing exactly which sources of leakage were addressed. In the Abstract, the pipeline is now described as “leakage-aware.” The Methods heading was revised to “Cross-validation design addressing sample-relatedness and feature-selection leakage,” and corresponding wording throughout the manuscript now refers specifically to the corrected sample-relatedness and feature-selection issues rather than implying complete immunity to leakage. Importantly, the Limitations section continues to state explicitly that selecting the best epoch by validation AUC on the same held-out fold may optimistically bias AUC estimates and remains an unresolved source of potential leakage; a fully nested cross-validation redesign is identified as future work. This change directly distinguishes corrected leakage sources from the residual epoch-selection limitation. Abstract, Methods (“Cross-validation design addressing sample-relatedness and feature-selection leakage”), Discussion/Limitations (first limitation), and Supporting Information S1 Fig caption.

Reviewer 2

R2.2: Claims of a graph-based predictive advantage should be toned down because the GAT–GCN difference is modest (mean AUC 0.624 vs. 0.599; paired p = 0.049), overall GAT performance was not statistically distinguishable from the matched permutation-derived null (p = 0.327), and some optimistic bias may remain. Addressed. We revised the Abstract, Results, Discussion, and Conclusion to present the predictive findings more cautiously and to separate the small GAT–GCN difference from the permutation-test result. The Abstract now states that GAT achieved a “slightly higher mean AUC” than GCN and immediately notes that GAT performance was not statistically distinguishable from the matched permutation-derived null. The Results heading now reads “Graph-based models showed modest predictive gains over classical machine-learning baselines,” and the repeated GAT–GCN result is described as a “small and statistically fragile difference.” The Discussion similarly states that repeated cross-validation showed only a small GAT–GCN difference, while the permutation section now explicitly states that the test “did not provide evidence that the observed classification performance exceeded the matched null distribution.” The Conclusion was also moderated to state that graph-based models showed modest predictive gains that were not confirmed against the permutation-derived null. Together, these revisions avoid presenting the observed performance differences as definitive evidence of a graph-based predictive advantage. Abstract (Results and Conclusions); Introduction (study questions); Results (“Graph-based models showed modest predictive gains…” and “Performance did not exceed a permutation-based null…”); Discussion (opening, model-comparison subsection, permutation subsection); Conclusion.

Attachment Submitted filename: Response_to_Reviewers_Round2_PONE-D-26-25663.docx

# Editor report: Decision Letter 2

**DOI:** 10.1371/journal.pone.0359656.r005

**Authors:** Feng Liu

16 Sep 2026

Rigorous validation of graph-based network analysis reappraises biologically coherent ADHD-associated transcriptomic modules in peripheral blood

PONE-D-26-25663R2

Dear Dr. Ali,

We’re pleased to inform you that your manuscript has been judged scientifically suitable for publication and will be formally accepted for publication once it meets all outstanding technical requirements.

Within one week, you’ll receive an e-mail detailing the required amendments. When these have been addressed, you’ll receive a formal acceptance letter and your manuscript will be scheduled for publication.

An invoice will be generated when your article is formally accepted. Please note, if your institution has a publishing partnership with PLOS and your article meets the relevant criteria, all or part of your publication costs will be covered. Please make sure your user information is up-to-date by logging into Editorial Manager at Editorial Manager® and clicking the ‘Update My Information' link at the top of the page. For questions related to billing, please contact billing support.

If your institution or institutions have a press office, please notify them about your upcoming paper to help maximize its impact. If they’ll be preparing press materials, please inform our press team as soon as possible -- no later than 48 hours after receiving the formal acceptance. Your manuscript will remain under strict press embargo until 2 pm Eastern Time on the date of publication. For more information, please contact onepress@plos.org.

Kind regards,

Feng Liu, Ph.D.

Academic Editor

PLOS One

Additional Editor Comments (optional):

Reviewers' comments:

# Editor report: Acceptance letter

**DOI:** 10.1371/journal.pone.0359656.r006

**Authors:** Feng Liu

PONE-D-26-25663R2

PLOS One

Dear Dr. Ali,

I'm pleased to inform you that your manuscript has been deemed suitable for publication in PLOS One. Congratulations! Your manuscript is now being handed over to our production team.

At this stage, our production department will prepare your paper for publication. This includes ensuring the following:

* All references, tables, and figures are properly cited

* All relevant supporting information is included in the manuscript submission,

* There are no issues that prevent the paper from being properly typeset

You will receive further instructions from the production team, including instructions on how to review your proof when it is ready. Please keep in mind that we are working through a large volume of accepted articles, so please give us a few days to review your paper and let you know the next and final steps.

Lastly, if your institution or institutions have a press office, please let them know about your upcoming paper now to help maximize its impact. If they'll be preparing press materials, please inform our press team within the next 48 hours. Your manuscript will remain under strict press embargo until 2 pm Eastern Time on the date of publication. For more information, please contact onepress@plos.org.

You will receive an invoice from PLOS for your publication fee after your manuscript has reached the completed accept phase. If you receive an email requesting payment before acceptance or for any other service, this may be a phishing scheme. Learn how to identify phishing emails and protect your accounts at https://explore.plos.org/phishing.

If we can help with anything else, please email us at customercare@plos.org.

Thank you for submitting your work to PLOS One and supporting open access.

Kind regards,

PLOS One Editorial Office Staff

on behalf of

Dr. Feng Liu

Academic Editor

PLOS One
